# Covalent Organic Frameworks on Cu_2_O Nanocubes as Rapid Proton/Electron Transfer Gates for Efficient NH_3_ Electrosynthesis from Nitrate in Neutral Media

**DOI:** 10.1021/jacs.5c16080

**Published:** 2025-12-23

**Authors:** Warisha Tahir, Yuqin Wei, Mao Wang, Islam E. Khalil, Prasenjit Das, Ting Wang, Chong Cheng, Shuang Li, Arne Thomas

**Affiliations:** † Department of Chemistry, Functional Materials, 26524 Technische Universität Berlin, 10623 Berlin, Germany; ‡ College of Polymer Science and Engineering, State Key Laboratory of Advanced Polymer Materials, 12530 Sichuan University, Chengdu 610065, China

## Abstract

Nitrate electroreduction to ammonia offers a dual opportunity: decarbonizing NH_3_ production by replacing the energy-intensive Haber-Bosch process and remediating nitrate-contaminated wastewater. While copper-based catalysts show promise for this transformation, their practical implementation is hindered by sluggish kinetics and competing hydrogen evolution pathways. Here, we report the integration of covalent organic framework (COF) layers onto a Cu_2_O surface, which enables rapid proton/electron transfer and substrate activation for efficient NH_3_ electrosynthesis from NO_3_^–^ in neutral media. By growing pyridine- or imidazole-decorated COF shells with varying thickness on Cu_2_O nanocubes, we achieve precise control over the microenvironment of the electrocatalyst surface. The pyridine-COF on Cu_2_O demonstrates 84% Faradaic efficiency for NH_3_ production with 92.11% selectivity and a record yield of 2.3 mg h^–1^ cm^–2^. In situ spectroscopic investigations reveal that the COF shells have multifunctional roles: they selectively transport reactants, stabilize key intermediates through hydrogen bonding interactions, and steer the reaction along an associative pathway that bypasses common side reactions. Our findings establish COF-gated core–shell architectures as a generalizable platform for designing efficient, selective, and durable electrocatalysts for broad applications.

## Graphical abstract

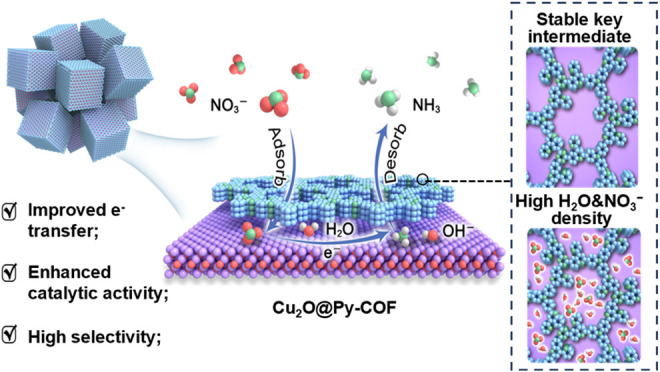

## Introduction

The accumulation of oxidative nitrogen species, particularly nitrate (NO_3_^–^), has emerged as a pressing environmental challenge. As major environmental pollutants, these compounds originate from industrial wastewater discharge, fossil fuel combustion, and intensive agricultural practices, collectively contributing to the global disruption of the natural nitrogen cycle.^1–3^ This anthropogenic interference now far exceeds planetary boundaries for nitrogen fixation, creating an urgent need for sustainable remediation strategies.^4,5^ Recent research has consequently focused on developing catalytic systems capable of not only capturing nitrate pollutants but also transforming them into valuable ammonia: a chemical of fundamental importance to modern agriculture and industry.^6–8^

Recently, nitrate electroreduction (eNO_3_RR) has acquired significant industrial interest as a sustainable alternative for ammonia (NH_3_) production. Unlike the energy-intensive and CO_2_-emitting Haber-Bosch process, the eNO_3_RR operates under ambient conditions and enables the direct NO_3_^–^ conversion into value-added NH_3_, offering a greener and more decentralized route to nitrogen fixation.^9,10^ Moreover, the dissociation energy of the N–O bond in eNO_3_RR (204 kJ/mol) is significantly lower than that of the N≡N bond in N_2_ (941 kJ/mol), making the process energetically more favorable.^11^ In neutral eNO_3_RR systems, the generation of NH_3_ strongly depends on the generation of active hydrogen (H*) and the efficient reduction of NO_3_^–^ to NO_2_^–^, which is often regarded as the rate-determining step. If the reaction rates of these two reactions are not well synchronized, this can result in the accumulation of intermediates and increased competitive hydrogen evolution (HER) as a side reaction. In recent years, a wide range of electrocatalysts, including precious metals such as platinum (Pt),^12^ silver (Ag),^13^ palladium (Pd),^14^ rhodium (Rh),^15^ as well as transition metals like nickel (Ni),^16,17^ cobalt (Co),^18,19^ iron (Fe),^20^ and copper (Cu),^21,22^ have been investigated as catalysts for eNO_3_RR. Among them, Cu-based materials stand out, due to their low cost, favorable surface charge distribution, excellent electrocatalytic activity, and strong affinity for nitrate-to-ammonia conversion.^23^ Moreover, the partially filled *d*-orbitals of Cu metal contribute to high exchange current densities and enhanced eNO_3_RR yield rate.^24^

Despite these advantages, the current benchmark Cu-based electrocatalysts still face significant challenges in achieving selective nitrate-to-ammonia conversion due to structural reconstruction during eNO_3_RR and the adsorption of various intermediates (H_2_, NH_2_NH_2_, and N_2_) on their surface, leading to poor intrinsic activity and low NH_3_ selectivity. To address these limitations, recent studies have focused on improving the eNO_3_RR performance of Cu-based electrocatalysts through surface modification.^25–28^ Another important consideration is that NO_3_^–^ conversion to NH_3_ proceeds via multiple hydrogenation steps, in which water dissociation serves as a key proton (H^+^) source. Accordingly, efficient eNO_3_RR requires rapid proton-electron transfer coupled with effective substrate activation at the catalyst surface.^29^ Unfortunately, Cu-based materials exhibit limited capability for the aforementioned processes, which therefore hinders their catalytic performance. Addressing these limitations requires catalyst architectures that simultaneously optimize three critical processes: nitrate adsorption, proton-coupled electron transfer, and ammonia desorption. An effective strategy involves encapsulating Cu-based materials within porous matrices that protect the catalyst against structural degradation, while maintaining efficient mass transport. Covalent organic frameworks (COFs) have emerged as particularly promising candidates for this purpose, offering precisely tunable chemical functionality combined with a well-defined porosity. Their modular design enables the creation of tailored microenvironments that can enhance both the activity and stability of embedded electrocatalysts for eNO_3_RR.

Here, to address the intrinsic limitations of conventional Cu-based materials, we report the *de novo* design of COF-gated Cu_2_O surfaces as highly efficient eNO_3_RR catalysts. Inspired by membrane-regulated biocatalytic systems, our approach integrates structural confinement with molecular-level gating to enable rapid proton and electron transfer and substrate activation. The COF shell mimics a cellular membrane by providing selective pathways, stabilizing NO_x_ intermediates, and regulating substrate access and product release, thereby enhancing electrochemical NO_3_^–^ reduction to NH_3_ in neutral media. The synthesis of the COF shells, which act as dynamic interfacial gate that regulate proton/electron transport and substrate accessibility, is achieved via a one-pot Povarov reaction, enabling stable pyridine- or imidazole-decorated COF shells, encapsulating Cu_2_O nanocrystals (named Cu_2_O@Py-COF and Cu_2_O@Im-COF, respectively). We demonstrate that the nitrogen-containing moieties (pyridine and imidazole) within the COF layers serve dual functions: stabilizing the Cu_2_O core and creating a locally polarized microenvironment rich in coordination sites. This engineered environment selectively modulates electron and proton transfer while stabilizing key NO_x_ intermediates through hydrogen bonding and Lewis acid–base interactions (Scheme 1). Structural characterization reveals an optimal parallel orientation of 2D COF layers relative to the Cu_2_O surface with aligned channels that enable efficient ion and mass transfer to active sites. The optimized Cu_2_O@Py-COF system demonstrates exceptional performance, achieving 2.3 mg h^–1^ cm^–2^ NH_3_ yield with 84% Faradaic efficiency at −0.7 V vs RHE while maintaining stability for at least 40 h. Furthermore, the systematic variation of the COF shell thickness from 25 to 75 nm reveals an optimum thickness at 35 nm, which provides the optimal balance between reactant accessibility and active site protection. In situ spectroscopic studies elucidate the reaction mechanism, showing how the COF microenvironment promotes an associative pathway in the eNO_3_RR for complete nitrate-to-ammonia conversion. This outstanding performance stems from the pyridine-functionalized COF shell, which stabilizes the Cu­(I) surface and facilitates the directed ions transport through the nitrogen coordination sites to active centers of the Cu_2_O core. This work establishes a generalizable platform for designing robust electrocatalysts that combine molecular precision with macroscopic performance for the sustainable electroreduction of nitrate pollutants to valuable NH_3_ under mild conditions.

**Scheme 1:**
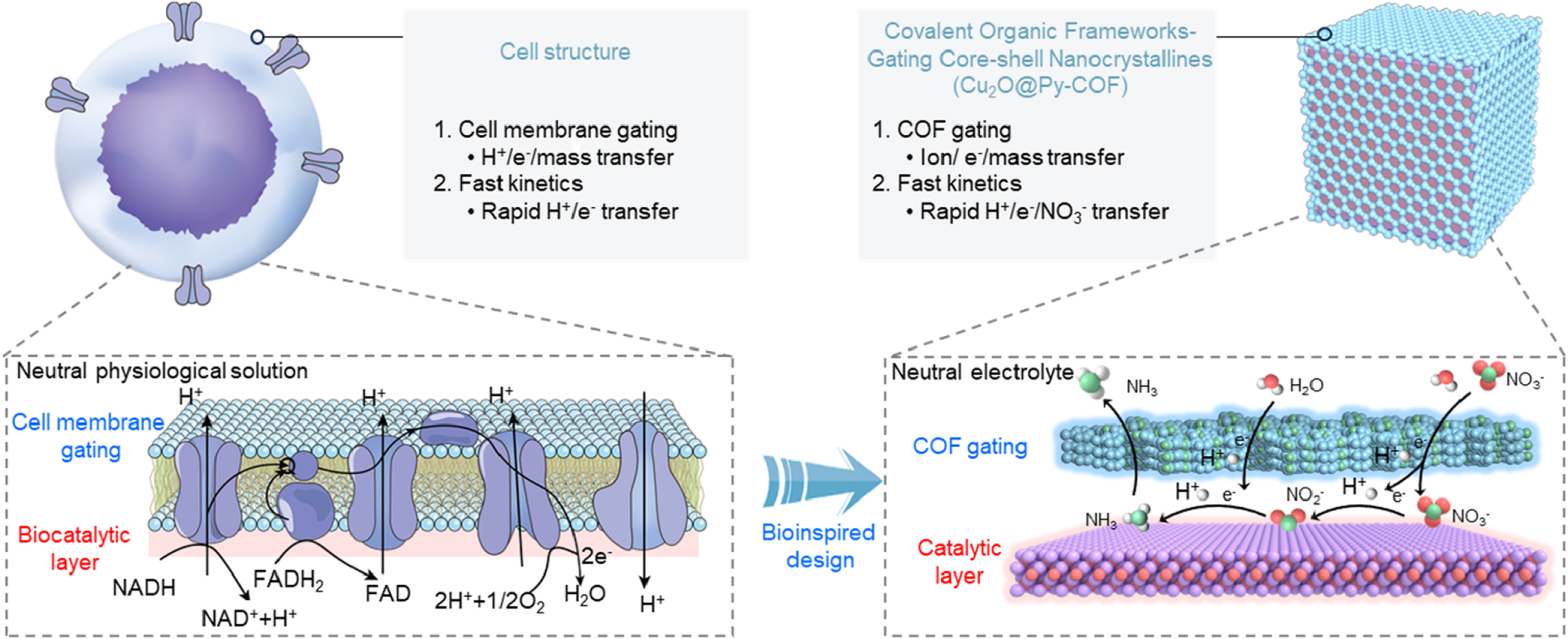
Bioinspired Design of Cu_2_O@Py-COF Structure Mimicking Cellular Catalysis

## Results and Discussion

To synthesize core–shell Cu_2_O@COF nanocrystals, first, Cu_2_O nanocubes (NCs) with well-defined (110) and (100) facets were prepared through a modified wet colloidal reduction approach, where *L*-ascorbic acid mediated the reduction of CuCl_2_ under controlled conditions (see SI Experimental Section).^30^ To precisely regulate the surface microenvironment of Cu_2_O, we developed a series of COF shells to satisfy three key criteria: (i) maintain structural integrity under reductive electrochemical conditions, (ii) ensure efficient ion transport to the Cu_2_O active sites, and (iii) incorporate tailored functional groups to modulate surface interactions. To achieve this, we employed a one-pot, multicomponent Povarov reaction (MCR) for COF synthesis, as it produces highly robust COFs with high crystallinity and well-defined porosity, allowing various functional groups to be incorporated into the pore interior.^31,32^ To prepare the MCR-COFs, amine and aldehyde functionalized building blocks, which conventionally form imine-linked COFs, are reacted in the presence of vinyl compounds to form quinoline linkages. Here, we employed 2-vinylpyridine and 1-vinylimidazole to construct MCR-COF shells with Lewis and Bronsted basic groups within the pore channels and investigated their influence on the ion transport, intermediate stabilization, and product selectivity during eNO_3_RR.

The Cu_2_O@COF-based core–shell architectures were constructed via a one-pot Povarov reaction, where presynthesized Cu_2_O NCs were introduced to a solution containing 2,4,6-tris­(4-aminophenyl)­triazine (TAT), 4,4′,4″-trinitrilotribenzaldehyde (TBA), and either 2-vinylpyridine or 1-vinylimidazole in a o-dichlorobenzene/n-butanol (1:1) solvent system, catalyzed by BF_3_·Et_2_O at 120 °C for 72 h (Figure 1a). This approach yielded two distinct systems: Cu_2_O@Py-COF and Cu_2_O@Im-COF, each preserving the cubic morphology of the Cu_2_O core while establishing a crystalline COF shell. Systematic variation of shell thickness was also achieved by adjusting the Cu_2_O NCs concentration while maintaining constant monomer quantities. The resulting materials are designated as Cu_2_O@Py-COF-y, where “y” shows the shell thickness in nanometers. The optimal 35 nm variant (Cu_2_O@Py-COF-35) was selected as the primary focus for detailed electrochemical characterization, with both thicker and thinner shells serving as a comparative benchmark to probe the structure-performance relationships. For clarity, the notation Cu_2_O@Py-COF refers specifically to Cu_2_O@Py-COF-35 throughout this work.

**Figure 1:**
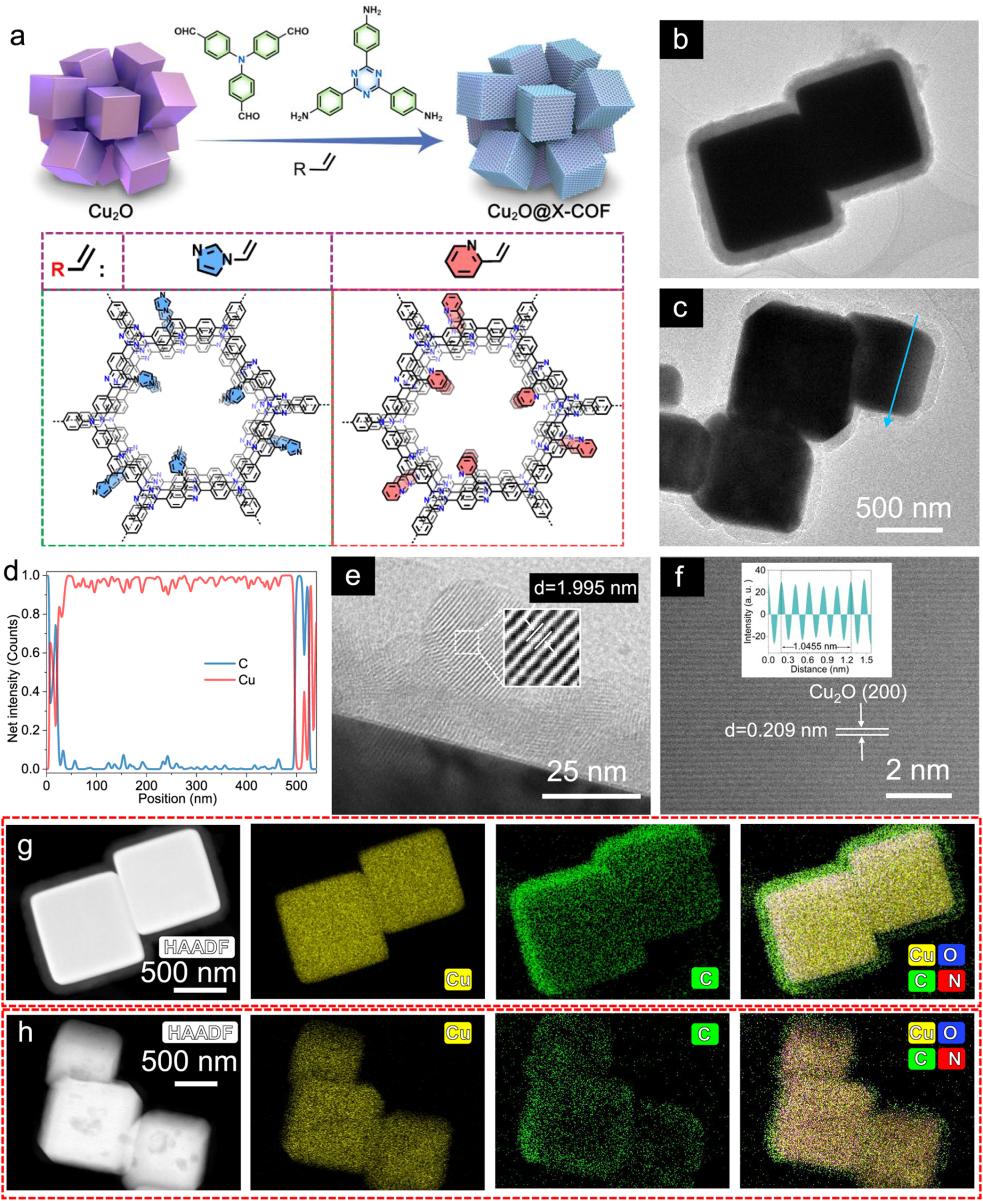
Synthesis and characterization of core–shell Cu_2_O@COFs structures. (a) Schematic illustration for the synthesis of MCR-COF shells encapsulating Cu_2_O NCs and chemical structure of the COF shells prepared by the Povarov reaction. STEM images of core–shell (b) Cu_2_O@Im-COF, and (c) Cu_2_O@Py-COF. (d) The corresponding STEM line scan measurement of core–shell Cu_2_O@Py-COF. (e) HRTEM images of the Py-COF shell around Cu_2_O NCs. (f) AC-TEM image and corresponding FFT pattern (inset) of core–shell Cu_2_O@Py-COF. HAADF-STEM EDS mapping images of core–shell (g) Cu_2_O@Im-COF and (h) Cu_2_O@Py-COF.

Microstructural characterization revealed the successful formation of well-defined core–shell architectures. High-resolution scanning transmission electron microscopy (STEM) demonstrated the epitaxial growth of crystalline COF shells along the Cu_2_O surfaces, maintaining the cubic symmetry and establishing distinct core–shell interfaces for both imidazole- and pyridine-functionalized variants (Figure 1b,c). Elemental line scanning across individual particles provided compelling evidence of the core–shell structure. The copper signal intensity peaked at the particle center and decayed radially, consistent with the Cu_2_O core dimensions. Conversely, the carbon distribution that represents the organic framework showed an inverse profile with the maximum intensity at the particle periphery (Figure 1d). This complementary elemental mapping unambiguously verifies the precise encapsulation of Cu_2_O cores within the functional COF shells, while the sharp interface between components suggests minimal interdiffusion or structural degradation during the coating process.

Advanced electron microscopy characterization provided atomic-scale insights into the core–shell architecture. High-resolution TEM images revealed well-ordered lattice fringes spanning multiple crystalline domains, confirming the high crystallinity of both the COF shell and the Cu_2_O core (Figure 1e). The measured spacing of 1.995 nm corresponds to the distance between the pore channels within the COF layers, consistent with powder X-ray diffraction (PXRD) data (see below). Aberration-corrected (AC−)­TEM analysis of pristine Cu_2_O NCs identified lattice spacings of 0.209 nm, which upon fast Fourier transformation (FFT) matched the (200) planes of cubic Cu_2_O (JCPDS 05–0667) (Figure 1f). The minor deviation from the reference value (0.213 nm) likely arises from the lattice or interfacial strain of Cu_2_O NCs components.^33^ Complementary high-angle annular dark-field (HAADF)-STEM imaging coupled with energy-dispersive X-ray spectroscopy (EDS) unequivocally demonstrated the spatial distribution of elements in both Cu_2_O@Im-COF and Cu_2_O@Py-COF systems (Figure 1g,h). SEM images of pure Cu_2_O show a well-defined cubic structure (Figure S1 a,b). The elemental maps showed clear segregation, with carbon signals localized to the shell region, while copper concentrated in the core region. Quantitative analysis revealed a uniform thickness of the COF shell (35 ± 2 nm) for Cu_2_O@Py-COF (Figure S1 c–f). Similar comprehensive morphological and structural characterizations are obtained for the Cu_2_O@Im-COF electrocatalyst with the shell thickness of 35 ± 2 nm (Figure S2). Control samples with varying shell thicknesses (25–75 nm) maintained the cubic morphology while exhibiting gradual changes in the COF coverage (Figures S3–S4), confirming the robustness of our synthetic approach for tailoring core–shell dimensions.

PXRD analysis provided a comprehensive structural verification of the electrocatalysts. The pristine Cu_2_O diffraction pattern exhibited characteristic peaks at 2θ = 29.6°, 36.3°, and 42.3°, which can be attributed to the (110), (111), and (200) planes of cubic Cu_2_O (JCPDS 05–0667), confirming phase purity and crystallinity (Figure 2a).^34^ All core–shell structures displayed additional low-angle reflections at 4.3 and 7.4°, corresponding to the (100) and (210) planes of the layered COF structure, demonstrating preserved periodicity in the organic shell.^35^ Systematic variation in shell thickness revealed a linear relationship between the COF diffraction intensity and shell thickness, with Cu_2_O peaks becoming more prominent when the shell is becoming thinner (Figure S5). Pawley refinement of the experimental data yielded unit cell parameters of pure Py-COF (*a* = *b* = 37 Å, *c* = 3.77 Å, α = β = 90°, γ = 120°) with excellent agreement factors (*R*_wp_ = 3.26%, *R*_p_ = 2.59%), while computational modeling confirmed an eclipsed AA stacking arrangement (Figure 2b). Similarly, the PXRD refinement of Im-COF (Figure S6) showed excellent agreement with the simulated pattern, further verifying the structural ordering of the imidazole-linked framework. All reflections of the pure COFs are also observed in the core–shell structures, albeit with lower intensities, as expected. The coexistence of distinct inorganic and organic diffraction features across all samples unambiguously confirms the successful integration of crystalline Cu_2_O cores with ordered COF shells.

**Figure 2:**
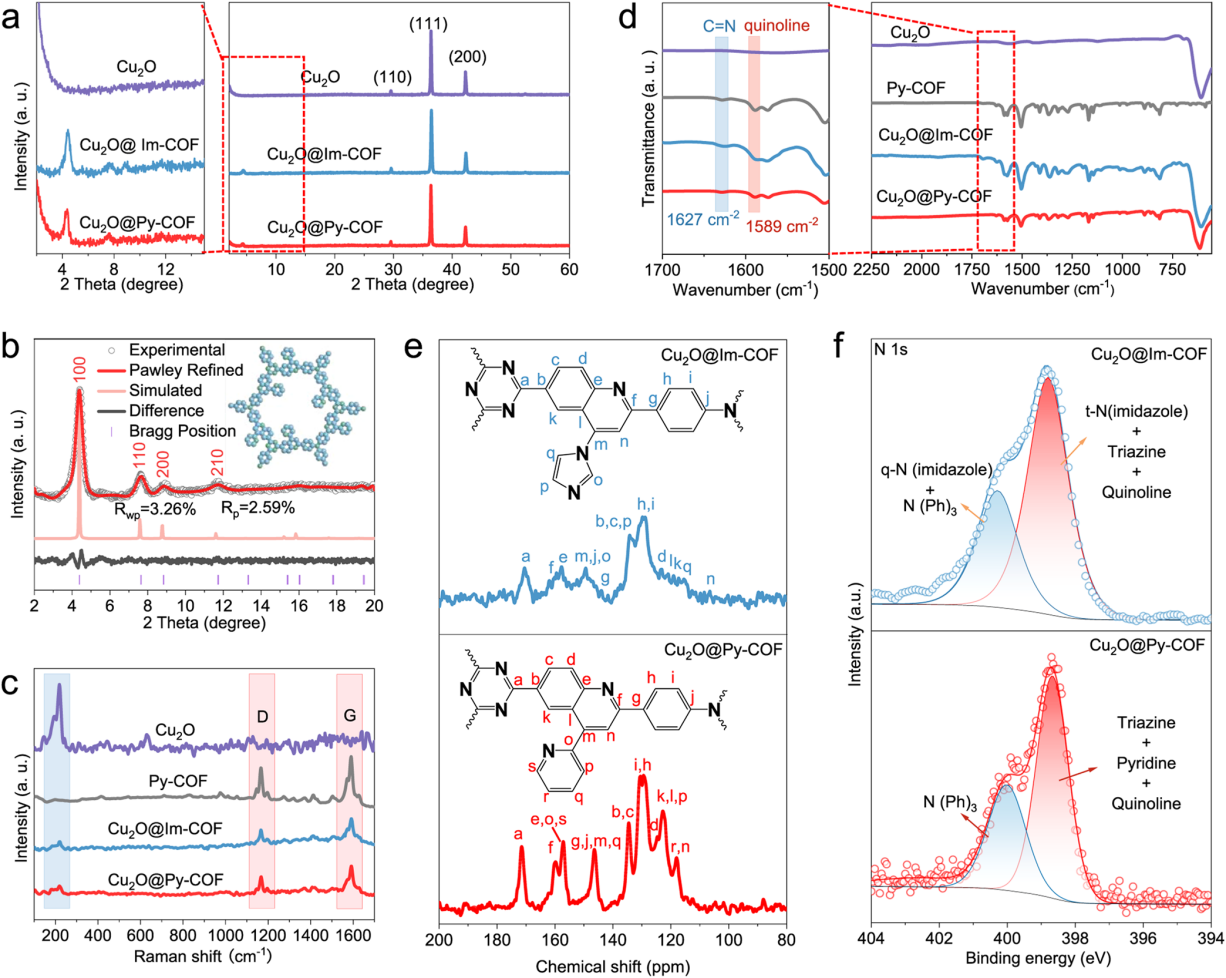
Characterization of Cu_2_O NCs and Cu_2_O@COF core–shell materials. (a) PXRD patterns of Cu_2_O NCs, Cu_2_O@Py-COF, and Cu_2_O@Im-COF. (b) The corresponding PXRD patterns for bare Py-COF: experimental (gray circle line), Pawley refined (red line), simulated pattern for AA stacking (yellow line), and Bragg positions (purple line). (c) FTIR (ATR) spectra of Cu_2_O NCs, Cu_2_O@Py-COF, and Cu_2_O@Im-COF. (d) Raman spectra and (e) solid-state ^13^C spectra and (f) N 1*s* XPS spectra of Cu_2_O@Py-COF and Cu_2_O@Im-COF.

Raman spectroscopic analysis revealed critical insights into the interfacial characteristics of the core–shell architectures. The characteristic Cu–O vibrational mode at 218 cm^–1^ in pristine Cu_2_O (consistent with literature reports)^36^ underwent significant broadening and a red shift upon COF encapsulation, providing direct evidence of strong electronic coupling between the inorganic core and organic shell.

The high-frequency region displayed two diagnostic bands: at 1168 cm^–1^ (C–N stretching in quinoline moieties) and 1594 cm^–1^ (combined C=N/C=C vibrations),^37–40^ both characteristic of the COF’s conjugated framework (Figure 2c). These spectral features remained remarkably consistent across all thickness variations (Cu_2_O@Py-COF-y), confirming structural preservation during shell thickness modulation (Figure S7).

Fourier transform infrared (FTIR) spectroscopy provided evidence of the formation of quinoline-linked COF shells and their interfacial interaction with Cu_2_O nanocubes across all core–shell variants. The characteristic vibrational signatures appeared in two distinct spectral regions: between 1500 and 2250 cm^–1^, the prominent absorption at 1627 cm^–1^ corresponds to C=N stretching from Schiff base linkages, while the 1590 cm^–1^ band arises from quinoline ring vibrations.^41–43^ Below 1500 cm^–1^, the sharp Cu–O lattice vibration at 607 cm^–1^ confirmed the preservation of the Cu_2_O core structure (Figure 2d). Remarkably, this vibrational fingerprint remained consistent across Cu_2_O@Py-COF with varying shell thicknesses (Figure S8), demonstrating that the synthetic protocol reliably produces intact core–shell architectures regardless of the shell thickness. The persistence of both organic (C=N) and inorganic (Cu–O) signatures in all samples verifies that the COF functionalization process maintains the structural integrity of both components while establishing well-defined organic–inorganic interfaces.

Complementary solid-state ^13^C NMR spectroscopy measurements elucidated the COF’s chemical structure. The spectrum shows characteristic resonances in the region between 110 and 170 ppm, which can be attributed to the aromatic carbons in the COF backbone. The peaks at 155 ppm (quinoline –C=N– quaternary carbons) in both frameworks verify the formation of conjugated networks through the Povarov reaction (Figure 2e). The narrow line widths and chemical shift consistency across all core–shell variants demonstrate the formation of uniform, well-defined COF coatings with preserved π-conjugation.^44^

X-ray photoelectron spectroscopy provided important insights into the evolution of the electronic structure from pristine Cu_2_O to Cu_2_O@COFs core–shell architectures. The N 1*s* spectra exhibited two well-resolved components at 398.7 eV (–C=N– in triazine/quinoline) and 400.1 eV (triphenylamine nitrogen), confirming again the preservation of COF connectivity upon integration (Figure 2f).^45^ Corresponding C 1*s* spectra revealed three distinct peaks: aromatic C=C (284.8 eV), C–N (285.6 eV), and C=N (286.8 eV), characteristic of the conjugated framework (Figures S9a and S10a).^46,47^ The Cu 2p region showed significant interfacial electronic effects. Pristine Cu_2_O displayed characteristic Cu^+^ signatures at 932.4 and 952.1 eV corresponding to Cu^+^ 2p_1/2_. Additionally, small shoulder peaks at 933.3 and 953.6 eV corresponding to the Cu^+^ 2p_3/2_ were observed. However, the presence of light shakeup satellite features (941–944 eV) confirms that the signal originates predominantly from Cu^+^ species rather than metallic Cu^0^ (with no satellite) in agreement with literature reports for Cu_2_O-based materials.^48–50^ Upon coating with COF shells, the Cu 2*p* binding energies shifted to a higher binding energy by ∼0.3 eV, implying the strong electronic interaction at the Cu_2_O@COF interface (Figures S9b, S10b, and S11a). Oxygen (O 1s) XPS analysis revealed dominant lattice oxygen (530.1 eV) with a secondary component (531.8 eV) corresponding to interfacial Cu–O–C linkages (Figures S9c, S10c, and S11b), while survey spectra confirmed elemental homogeneity across all samples (Figure S12).^51^ Together, these spectroscopic techniques establish that the synthetic protocol yields structurally intact core–shell systems with strong interfacial electronic interactions, while maintaining the distinct chemical identity of each component.

Thermogravimetric analysis under oxidative conditions provided quantitative insights into the composition and thermal behavior of the core–shell architectures. Pristine Cu_2_O NCs exhibited the expected 10% mass gain at 300 °C, corresponding to complete oxidation to CuO. The Cu_2_O@Im-COF showed a markedly reduced net mass gain (∼4.5%), reflecting competing processes of Cu_2_O oxidation and imidazole-COF decomposition. For Cu_2_O@Py-COF, the same competing processes are observed, however, yielding an overall weight loss of ∼8%. This weight loss is more pronounced for the samples with thicker (Cu_2_O@Py-COF-75) and less pronounced for the sample with thinner shell (Cu_2_O@Py-COF-25), following the expected trend for samples with varying amounts or organic and inorganic components (Figures S13–S14).

### Electrocatalytic NO_3_RR Performance

The nitrate electroreduction performance was evaluated in a three-electrode system under ambient conditions in neutral media. It is noteworthy that Cu_2_O exhibits lower intrinsic conductivity than metallic Cu; its semiconducting nature and cubic morphology confer unique catalytic advantages. The exposed {100} facets of cubic Cu_2_O present ordered Cu^+^ surface sites with optimized electronic density, enabling selective NO_3_^–^ adsorption and stepwise reduction. Following activation in 0.1 M Na_2_SO_4_ electrolyte, linear sweep voltammetry (LSV) revealed distinct catalytic responses upon introducing 0.1 M NaNO_3_ as the nitrate source. Both pristine Cu_2_O and Cu_2_O@COF core–shell composites exhibit substantial current density enhancements in nitrate-containing electrolytes, confirming efficient nitrate reduction. Notably, the Cu_2_O@Py-COF system demonstrated superior catalytic activity, achieving the lowest onset potential (−0.25 V vs RHE) and highest current density among all variants (Figure 3a).

**Figure 3:**
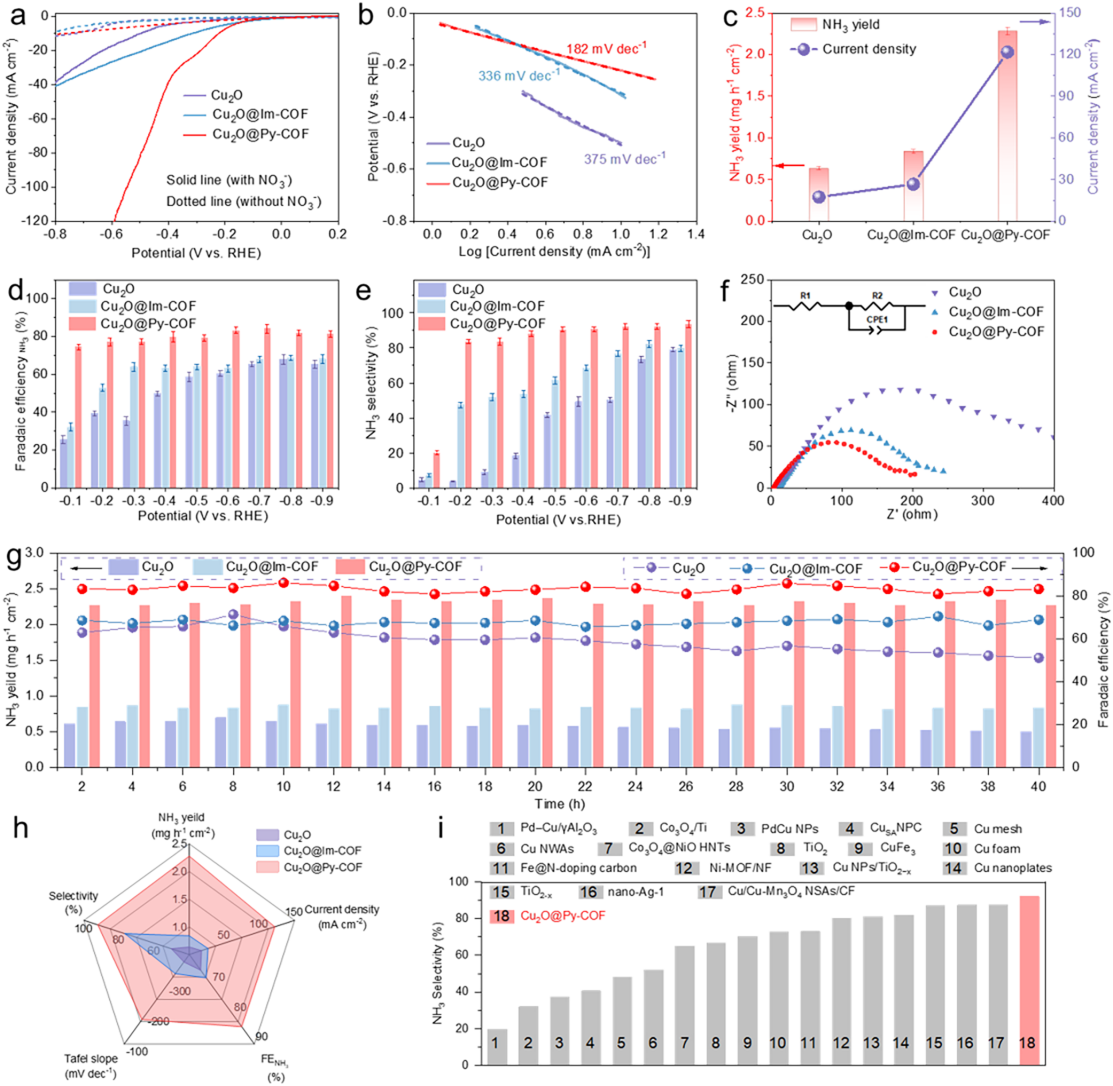
eNO_3_RR performance of Cu_2_O@COF core–shell electrocatalysts. (a) LSV curves in Ar saturated 0.1 M Na_2_SO_4_ without (dashed line) and with (solid lines) NaNO_3_ (catalyst loading 1 mg cm^–2^ and reaction temperature 25 °C) at scan rate of 10 mVs^–1^, (b) Tafel plots at different potentials. (c) Comparison of NH_3_ yield rate (histogram) and current density (line) measured at −0.7 V vs RHE. (d) Potential-dependent Faradaic efficiency and (e) nitrogen selectivity. (f) EIS Nyquist plots (amplitude: 10 mV, open-circuit potential, frequency range: 100 kHz–0.1 Hz) of the pristine Cu_2_O NCs, Cu_2_O@Py-COF, and Cu_2_O@Im-COF electrocatalysts. (g) Stability tests of pristine Cu_2_O NCs, Cu_2_O@Im-COF, and Cu_2_O@Py-COF at −0.7 V versus RHE for 40 h under ambient conditions. (h) Comparison Rader plot of the electrochemical NO_3_RR performance of Cu_2_O@Py-COF, Cu_2_O@Im-COF, and pristine Cu_2_O NCs. (i) Comparison of electrochemical NO_3_RR selectivity for Cu_2_O@Py-COF with recently reported electrocatalysts.

To gain insights into the electrochemical kinetics of the eNO_3_RR, Tafel slopes were calculated from LSV data for pristine Cu_2_O NCs and Cu_2_O@COFs core–shell electrocatalysts. All the synthesized electrocatalysts exhibited Tafel slopes exceeding 120 mV dec^–1^, suggesting that the rate-determining step (RDS) involves the electron transfer process from adsorbed nitrate (*NO_3_^–^) to nitrite (*NO_2_^–^), consistent with previous reports.^52^ Considering the continuous reaction pathway, the formation of NO_2_^–^ (nitrite) is a critical intermediate step in the overall eNO_3_RR process. Among the materials, Cu_2_O@Py-COF demonstrated the lowest Tafel slope of 182 mV dec^–1^, indicating faster electron transfer and more favorable reaction kinetics compared to Cu_2_O@Im-COF (335 mV dec^–1^) and pristine Cu_2_O NCs (375 mV dec^–1^) (Figure 3b).

Therefore, a comprehensive evaluation of the NH_3_ yield and corresponding current densities was conducted. Among all electrocatalysts, Cu_2_O@Py-COF exhibited the highest NH_3_ yield rate of 2.3 mg h^–1^ cm^–1^ along with a maximum current density of ∼121 mA cm^–2^, highlighting its superior activity for eNO_3_RR. In contrast, Cu_2_O@Im-COF showed just moderate enhancement in performance over pristine Cu_2_O NCs (Figure 3c). To optimize the electrochemical performance of the Cu_2_O@COFs core–shell electrocatalysts for nitrate reduction, the Faradaic efficiency and selectivity for NH_3_ production were evaluated across different potential ranges from −0.1 to −0.9 V vs RHE, revealing a characteristic volcano-type trend. Among all catalysts, Cu_2_O@Py-COF again consistently exhibited the highest Faradaic efficiency and selectivity across the entire range, reaching a maximum of FE 84% with 92.11% NH_3_ selectivity at −0.7 V (Figure 3d,e). To further confirm the selectivity of the NO_3_^–^ reduction pathway, quantitative analyses of all nitrogen-containing byproducts (NO_2_^–^ and N_2_H_4_) were performed (Figure S15). NH_3_ was identified as the dominant product, accompanied by minor amounts of NO_2_^–^ and negligible hydrazine (N_2_H_4_) formation, consistent with an eight-electron reduction pathway. At more negative potentials beyond −0.7 V, the Faradaic efficiency values for all electrocatalysts declined primarily due to the increased rate of hydrogen evolution, competing with the eNO_3_RR. Furthermore, electrochemical impedance spectroscopy (EIS) was performed to assess the charge transfer resistance of each electrocatalyst. The EIS Nyquist plot of Cu_2_O@Py-COF exhibited the smallest semicircle diameter, indicative of lower ohmic charge transfer resistance and enhanced ionic conductivity compared to those of Cu_2_O@Im-COF and pristine Cu_2_O NCs (Figure 3f).

Apart from the catalytic activity, the long-term electrochemical stability of the catalysts was examined at −0.7 V vs RHE. Remarkably, the Cu_2_O@Py-COF maintained its catalytic activity over 20 consecutive cycles (40 h). Throughout the stability test, the NH_3_ yield remained consistently high at 2.3 mg h^–1^ cm^–2^, with a stable Faradaic efficiency of 84%. In comparison, Cu_2_O@Im-COF retained ∼90% of its initial current density, while pristine Cu_2_O showed a more pronounced decline, confirming the stabilizing influence of the COF shells (Figure 3g). These results indicate the robust catalytic performance and structural integrity of the Cu_2_O@Py-COF electrocatalyst under prolonged electrochemical conditions. The superior durability of Cu_2_O@Py-COF is attributed to the strong interfacial coordination between the pyridine moieties and Cu_2_O, which suppresses leaching and structural reconstruction during extended electrolysis. These results collectively highlight the structural robustness and interfacial integrity of the Cu_2_O@COF core–shell architectures. Furthermore, to assess the pH-dependent versatility of the Cu_2_O@Py-COF catalyst, additional LSV and Faradaic efficiency measurements were performed under strongly acidic (pH 0–1), neutral (pH 7), and strongly alkaline (pH 13–14) conditions (Figure S16). The catalyst maintained appreciable activity across the full pH range, with the highest FE and minimal hydrogen evolution observed at pH 7. The superior neutral-pH performance can be ascribed to the enhanced structural stability of Cu_2_O and the optimal balance between proton availability and nitrate adsorption, whereas extreme pH environments induce either Cu_2_O reduction (acidic) or electrostatic repulsion of NO_3_^–^ ions (alkaline). These results highlight the intrinsic robustness of Cu_2_O@Py-COF and its relevance for nitrate remediation under environmentally neutral conditions.

In addition, the quinoline-linked COF framework, formed via the Povarov reaction, includes a complete set of aromatic C–C and C–N bonds, which remain chemically inert under the electrochemical NO_3_RR conditions. The negligible changes in activity and Faradaic efficiency over 40 h further confirm the structural integrity and interfacial robustness of the Cu_2_O@COF architectures. In contrast, the pristine Cu_2_O catalyst showed a noticeable decline in performance, with a 20% Faradaic efficiency loss and a reduced NH_3_ yield over the same duration, which is likely due to surface degradation, partial dissolution, and deactivation of active sites under continuous operation.

These findings establish that the optimal operating potential of −0.7 V enables selective and efficient NH_3_ production. The superior performance of Cu_2_O@Py-COF is attributed to the N-rich pyridine units in the COF shell with 35 nm thickness, which offer abundant active sites for strong NO_3_^–^ adsorption and facilitate its subsequent reduction to NH_3_ (Figure 3 h). Notably, the eNO_3_RR values of Cu_2_O@Py-COF represent the highest among recently reported Cu-based eNO_3_RR electrocatalysts (Figure 3i and SI Table 1).

Core–shell materials with varying shell thickness (Cu_2_O@Py-COF-y) similarly showed a pronounced current response to nitrate introduction, though with varying intensity depending on the shell dimensions (Figures S17–S18). These systematic measurements establish clear structure–activity relationships while validating the core–shell design principle for efficient nitrate electroreduction. In comparison, both the thicker shell Cu_2_O@COF-75 and thinner shell Cu_2_O@COF-25 catalysts exhibited slightly higher Tafel slopes of 201 and 203 mV dec^–1^, respectively (Figure S19). These results further highlight that an optimal COF shell thickness is critical to facilitate ion transport while preserving efficient charge transfer at the Cu_2_O active sites. Furthermore, electrochemical impedance spectroscopy (EIS) was performed to assess the charge transfer resistance of each catalyst. The Nyquist plots were recorded at open-circuit potential with an amplitude of 10 mV over the frequency range of 100 kHz to 0.1 Hz. The EIS Nyquist plot of Cu_2_O@Py-COF exhibited the smallest semicircle diameter, indicative of lower ohmic charge transfer resistance and enhanced ionic conductivity compared to Cu_2_O@COF-75 and Cu_2_O@COF-25 (Figure S20). When considering the effect of the thickness of the COF shell, both the thicker Cu_2_O@COF-75 and thinner Cu_2_O@COF-25 showed lower NH_3_ yields of 1.75 and 1.5 mg h^–1^ cm^–1^, respectively (Figure S21). These results further underscore the importance of the optimized COF shell thickness for achieving efficient NO_3_RR performance. Finally, Cu_2_O@COF-75 and Cu_2_O@COF-25 electrocatalysts showed lower NH_3_ current density with lower FE and NH_3_ selectivity at −0.7 V (Figure S22).

Mechanistic insights into the eNO_3_RR pathway were obtained through *in situ* spectroscopic investigations. In situ Raman spectroscopy under operational conditions (−0.7 V vs RHE) revealed two key intermediates (Figure 4a): a band at 1150 cm^–1^ corresponding to adsorbed *NH_2_ species, which arises from the hydrogenation of nitrite-derived intermediates, and another at 1523 cm^–1^ attributed to the crucial *NH intermediate for the eNO_3_RR pathway to NH_3_.^53^ To confirm the nitrogen source during the eNO_3_RR, ^1^H NMR spectra were recorded using both ^14^NO_3_^–^ and isotopically labeled ^15^NO_3_^–^ as reactants. The calibration curve was first established by using standard solutions of (^14^NH_4_)_2_SO_4_ and (^15^NH_4_)_2_SO_4_. Following the eNO_3_RR electrolysis, containing 0.1 M of ^14^NO_3_^–^ or ^15^NO_3_^–^ at −0.7 V vs RHE, a distinct triplet signal for ^14^NH_4_^+^ and a doublet corresponding to ^15^NH_4_^+^ were observed in the NMR spectra, which precisely matched their respective ammonium sulfate standards (Figure 4b).

**Figure 4:**
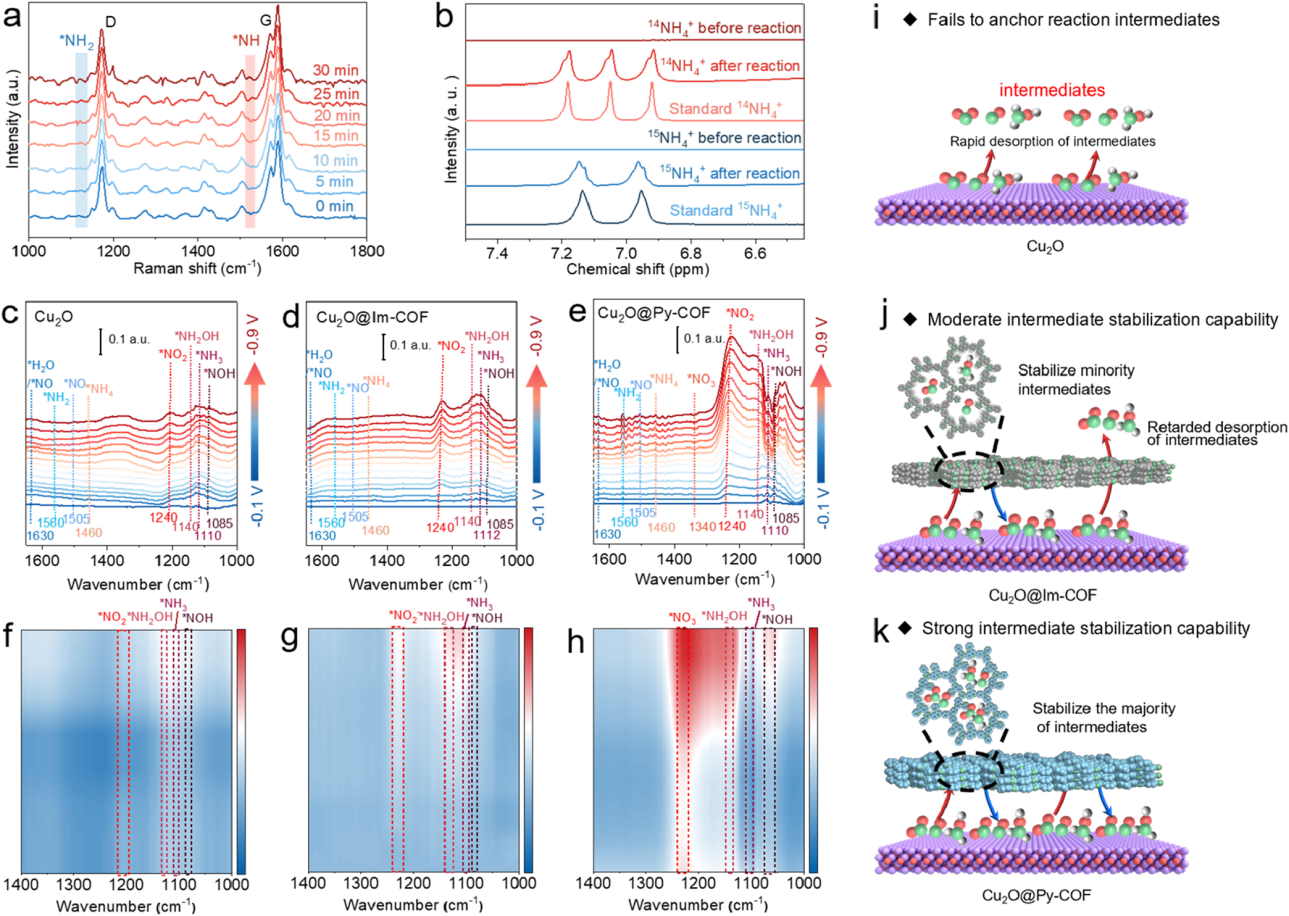
In situ spectroscopic analysis on the electrochemical eNO_3_RR process. In situ (a) Raman spectra of Cu_2_O@Py-COF and (b) ^1^H NMR spectra using ^15^NO_3_^–^ as the nitrogen source of Cu_2_O@Py-COF before and after electrolysis. *In situ* FTIR spectra acquired at different applied potentials (from −0.1 to −0.9 V vs RHE) for (c) Cu_2_O, (d) Cu_2_O@Im-COF, and (e) Cu_2_O@Py-COF. Corresponding 2D FTIR contour plots for (f) Cu_2_O, (g) Cu_2_O@Im-COF, and (h) Cu_2_O@Py-COF electrocatalyst for eNO_3_RR. Scheme of the proposed mechanism for NO_3_RR on (i) Cu_2_O, (j) Cu_2_O@Im-COF, and (k) Cu_2_O@Py-COF.

This definitive isotopic tracing confirms that the produced ammonia derives exclusively from the nitrate reactant rather than other potential nitrogen sources, especially not from decomposition of the COF shells, validating the catalytic specificity of the core–shell architecture.

*In situ* FTIR spectroscopy at −0.7 V vs RHE provided molecular-level resolution of the nitrate reduction pathway on pristine Cu_2_O NCs, Cu_2_O@Im-COF, and Cu_2_O@Py-COF surfaces. On bare Cu_**2**_O NCs, only weak bands corresponding to *NO_2_ and *NH_4_^+^ species were observed, indicating inefficient NO_3_^–^ conversion (Figure 4c). On the other hand, the time-dependent spectra of Cu_2_O@Im-COF revealed that the intensity of key intermediates such as *NH_2_OH and *NH_4_^+^ was higher, suggesting an improved NO_3_^–^ adsorption and hydrogenation due to the presence of the COF layer (Figure 4d). Notably, a variety of nitrogenous intermediates was observed on Cu_2_O@Py-COF, seen on more prominent and earlier-appearing bands which can be attributed to nitrate (NO_3_^–^ at 1340 cm^–1^), nitrite (NO_2_^–^ at 1240 cm^–1^) and adsorbed *NO (1505 cm^–1^) to various hydrogenated species including *NOH (1086 cm^–1^), *NH_2_OH (1140 cm^–1^), and *NH_2_ (1560 cm^–1^), ultimately yielding NH_4_^+^ (1460 cm^–1^) as the terminal product (Figure 4e). This confirms that the pyridine-functionalized COF shell significantly enhances the catalytic progression of NO_3_^–^ to NH_3_ by stabilizing reactive intermediates. Notably, a broad band at 1630 cm^–1^ is associated with H–O–H bending, likely due to water or hydrated *NO species present in the electrolyte environment. Besides, the peaks at 1110 cm^–1^ are ascribed to adsorbed *NH_3_. The increasing intensity of the *NH_3_ band over time indicates the formation and accumulation of ammonia as the final product of the eNO_3_RR process.^54^

To investigate the evolution of surface-bound species in greater detail, 2D FTIR contour maps were constructed. The Cu_**2**_O NCs exhibit weak and faint contour features, suggesting unstable adsorption of reaction intermediates (Figure 4f). In contrast, the Cu_2_O@Im-COF displays moderately enhanced signals corresponding to *NO_2_ and *NH_2_OH intermediate species (Figure 4g), indicating improved, yet limited, intermediate stabilization. Notably, the significantly stronger and more extensive contour features corresponding to successive intermediates (*NO_3_^–^, *NO_2_^–^, *NO, *NOH, *NH_2_OH, and *NH_3_) are shown on the Cu_2_O@Py-COF surface (Figure 4h). Combined with in situ Raman spectroscopy, the data support an associative reaction pathway, wherein nitrate reduction proceeds via sequential proton-coupled electron transfers (PCET) steps rather than through direct N–O bond cleavage. The Py-COF shell plays a pivotal role in this process: its nitrogen-rich architecture offers multiple hydrogen bonding sites that not only stabilize key intermediates but also facilitate efficient proton and electron delivery. This dual functionality lowers the activation barriers for successive hydrogenation steps while effectively suppressing competing pathways, resulting in the system’s exceptional selectivity toward NH_3_. Together, these spectroscopic insights demonstrate how precisely tailored organic–inorganic hybrid interfaces can direct complex electrocatalytic processes by regulating the transformations through the controlled stabilization of transition states and reaction intermediates.

The mechanistic differences among Cu_2_O, Cu_2_O@Im-COF, and Cu_2_O@Py-COF are illustrated in Figure 4i–k. In the case of pristine Cu_2_O, the surface fails to effectively anchor NO_3_RR intermediates such as NO_2_^–^, NO, and NH_2_OH, leading to rapid desorption and low surface coverage. This insufficient interaction results in poor stabilization of reactive species, hindering the stepwise hydrogenation pathway and ultimately lowering both the selectivity and NH_3_ yield rate (Figure 4i). Meanwhile, the introduction of Im-COF shell on Cu_2_O@Im-COF provides a more structured and chemically interactive surface, allowing moderate intermediate adsorption and stabilization while slowing down their desorption. However, only a minority of the intermediates are effectively retained due to the relatively weaker dipolar interactions and limited electron-donating ability of the imidazole framework (Figure 4j). In contrast, the most favorable adsorption and desorption dynamics are observed by Cu_2_O@Py-COF, where a nitrogen-rich Py-COF shell microenvironment strongly coordinates nitrogen intermediates, particularly NO and NH_2_OH, thus facilitating sequential proton-coupled electron transfer steps and enabling efficient NH_3_ production. Additionally, the controlled desorption of the final NH_3_ product ensures both high selectivity and catalyst turnover (Figure 4k). The combined results highlight the pivotal role of the chemical nature of the COF shell in directing the adsorption or desorption dynamics and finally determining the overall catalytic performance.

To elucidate the role of the COF layers in selective ion and molecule transport, all-atom molecular dynamics (MD) simulations were conducted. Two three-layered COF membranes divided the simulation box into interlayer, within-layer, and out-of-layer regions (Figure 5a and Methods). NO_3_^–^ and Na^+^ ions were randomly placed in the out-of-layer region, while NH_3_ molecules were confined to the interlayer. All of the species were then allowed to diffuse under a concentration gradient. Py-COF shows a higher NO_3_^–^ affinity compared to Im-COF. After 20 ns of diffusion, only 27% of NO_3_^–^ ions remained in the out-of-layer region for Py-COF, whereas a significantly higher residual fraction (57%) was observed for Im-COF (Figure 5b). A more detailed analysis revealed that 62% of NO_3_^–^ ions were absorbed within the Py-COF membrane, in contrast to only 11% for Im-COF, indicating a strong affinity of Py-COF for NO_3_^–^ ions. This preferential binding was further validated by analyzing the interaction energies between the COF membranes and NO_3_^–^ ions (Figure 5c). Decomposition of the interaction energies showed that Py-COF exhibited stronger van der Waals (vdW) and electrostatic interactions (Figure 5d). Electrostatic potential (ESP) surface analysis indicated that the pyridine groups in Py-COF displayed more positive electrostatic potential regions, which favor electrostatic interactions with NO_3_^–^ compared to Im-COF (Figure 5e). Furthermore, molecular dynamics (MD) simulations were performed to elucidate the influence of the COF shell thickness on ions diffusion. Models with one, three, and six COF layers were constructed to represent progressively thicker shells. However, an excessively thick COF membrane markedly hinders ion penetration and diffusion through the channels (Figure S23). This observation aligns well with the experimental trend, emphasizing that an optimal COF thickness is essential to maintain a favorable balance between the NO_3_^–^ accessibility and transport efficiency across the COF interface. Note that in these simulations, a slightly higher NaNO_3_ concentration (∼0.5 M) than in the experiments (0.1 M) was used to accelerate ion dynamics and improve sampling statistics. This adjustment does not affect the relative ion-COF interaction trends or the mechanistic interpretation. Dipole moment calculations further revealed that Py-COF exhibits a significantly higher dipole moment (1.51 D) than Im-COF (0.68 D), which may also enhance the interactions between the NO_3_^–^ ions and the pyridine groups of Py-COF. Given the close interface between the COF shell and Cu_2_O nanocrystals, this specific affinity effectively enriches local NO_3_^–^ concentration, thereby facilitating the eNO_3_RR. In addition to NO_3_^–^ ions, protons originating from water molecules also play a crucial role in the reaction. Py-COF exhibited weaker hydrogen bonding interactions with water, as evidenced by the smaller number and longer bond lengths of hydrogen bonds formed (Figure 5f). This weak interaction allows water molecules to diffuse more freely through Py-COF channels to reach the Cu_2_O surface. Notably, we also found that Py-COF accommodated a greater number of water molecules compared to Im-COF (Figure 5g), which may enhance the local proton supply and lead to fast proton transport, essential for the catalytic process. We speculate that the higher dipole moment of Py-COF facilitates stronger dipole–dipole interactions with water molecules, contributing to this effect. These simulation results collectively confirm that the strong NO_3_^–^ affinity of Py-COF, combined with its superior water accommodation capability and enhanced proton availability, makes it highly effective at enriching reactants and accelerating the NO_3_RR reaction rate (Figure 5h).

**Figure 5:**
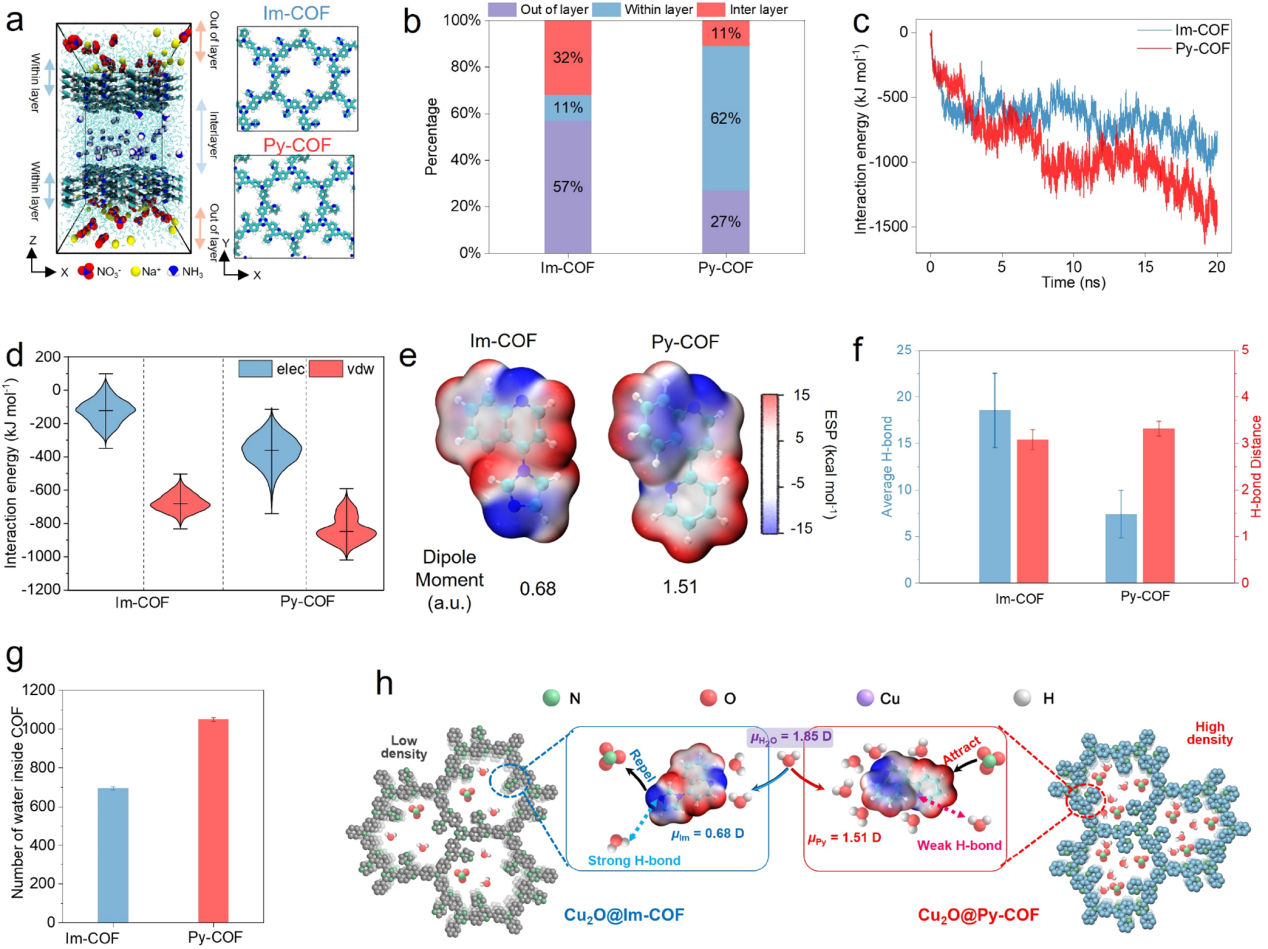
Impact of COF shells on selective ion and molecule transport. (a) MD simulation setup with three-layer COF membranes dividing the simulation box into three regions. The system is filled with water; NO_3_^–^ and Na^+^ are placed in the outer regions, and NH_3_ molecules are positioned in the central region (Color code: oxygen-red; carbon-cyan; hydrogen-white; nitrogen-blue; Na^+^-yellow). (b) Percentage of NO_3_^–^ in different regions after 20 ns. (c) Time-dependent interaction energy between Im/Py-COFs and NO_3_^–^. (d) Decomposition of Im/Py-COFs NO_3_^–^ interactions into electrostatic (elec) and van der Waals (vdW) components. (e) electrostatic potential (ESP) surfaces and dipole moments of Py-COF and Im-COF. (f) Average number and bond length of hydrogen bonds formed between water molecules and Im/Py-COFs. (g) Average number of water molecules confined within the Im/Py-COFs channels. (h) Illustration of how Py-COF facilitate NO_3_^–^ reduction. The strong NO_3_^–^ affinity, enhanced water accommodation and increased proton availability synergistically contribute to efficient reactant enrichment and accelerated NO_3_RR kinetics.

## Conclusion

This work presents a rational strategy for designing functional COF layers on Cu_2_O surfaces to enable efficient proton and electron transfer and substrate activation, achieving superior electrochemical nitrate (NO_3_^–^) reduction to NH_3_ in neutral media. By engineering pyridine (Cu_2_O@Py-COF) and imidazole (Cu_2_O@Im-COF) interfaces, we constructed a modular platform that simultaneously addresses three key challenges in nitrate electroreduction (eNO_3_RR): catalyst stability, reaction intermediate stabilization, and reactant accessibility. The COF shells not only serve as protective barriers against surface degradation of Cu_2_O but also act as an active interfacial layer that regulates ion transport, stabilizes critical eNO_3_RR key intermediates through electronic and hydrogen bonding interactions, and further promotes selective access of NO_3_^–^ ions to the Cu_2_O active sites. The optimized Cu_2_O@Py-COF system exhibits exceptional performance, achieving an NH_3_ production rate of 2.3 mg h^–1^ cm^–2^ with 84% Faradaic efficiency while maintaining operational stability over 40 h of continuous operation. In situ spectroscopic analysis, including FTIR and Raman spectroscopy combined with isotopic labeling, elucidates an associative reaction mechanism where sequential proton-coupled electron transfers convert *NO_3_^–^ to *NH_3_ without disruptive N–O bond cleavage. Crucially, we identify an optimal COF shell thickness (∼35 nm), which effectively balances the electron transfer efficiency with the mass transport of reactants and products. These findings underline the transformative role of precisely engineered organic–inorganic interfaces that can transform conventional electrocatalysts into high-performance systems. The design principles developed in this study, combining molecular-level functionality with nanoscale structure control, provide a generalizable approach for developing efficient, durable, and selective catalysts for sustainable ammonia synthesis and other complex electrochemical transformations.

## Supplementary Material



## Notes

Additional experimental details, materials, methods, and additional characterization (SEM, HRTEM, PXRD, Pawley refinement, Raman, FTIR, XPS, TGA) (PDF)
