# Supplementary material for: Covalent Organic Frameworks on Cu2O Nanocubes as Rapid Proton/Electron Transfer Gates for Efficient NH3 Electrosynthesis from Nitrate in Neutral Media
Source: J Am Chem Soc. 2025 Dec 23;148(1):743–55. doi: 10.1021/jacs.5c16080 (PMC12814341; doi:10.1021/jacs.5c16080)
Supplement: Supplementary file 1 [file ja5c16080_si_001.pdf]

Supporting Information for

Covalent Organic Frameworks on Cu_2O Nanocubes as Rapid Proton/Electron Transfer Gates for Efficient NH_3 Electrosynthesis from Nitrate in Neutral Media

Warisha Tahir,[1,#] Yuqin Wei,[2,#] Mao Wang,[2] Islam E. Khalil,[1] Prasenjit Das,[1] Ting Wang,[2] Chong Cheng,[2] Shuang Li,[2,*] and Arne Thomas,[1,*]

[1] Department of Chemistry Functional, Materials, Technische Universität Berlin, 10623 Berlin, Germany

[2] College of Polymer Science and Engineering, State Key Laboratory of Advanced Polymer Materials, Sichuan University, Chengdu, 610065, China

[#] These authors contributed equally to this work.

E-mail: (S. Li) shuang.li@scu.edu.cn; (A. Thomas) arne.thomas@tu-berlin.de

Section 1. General Materials and Methods

Materials: The primary reagents used in this study include 1,2-dichlorobenzene (o-DCB, 99%), anhydrous n-butanol (n-BuOH, 99%), copper (II) chloride ($CuCl_2$), boron trifluoride-diethyl ether (BF_3•OEt_2), 2-vinyl pyridine, and L-ascorbic acid were purchased from Sigma Aldrich Chemicals. 4,4',4"(1,3,5-triazine-2,4,6-triyl)trainline (95%), and 4,4',4"-trinitrilotribenzaldehyde were all supplied by BLD pharm. Acetic acid (>99.0%), methanol (MeOH), and ethanol (EtOH) were purchased from Carl Roth. Sodium hydroxide (NaOH) and 2-vinylpyridine were purchased from Thermo Fisher Scientific. All chemicals were of analytical grade and used without further purification. Deionized water used during the material synthesis and for conducting experiments was purified using a Direct-Q 3UV water purification system (Millipore Corp., France).

X-ray powder diffraction (XRD) patterns were collected on a Haoyuan multifunctional DX-2700BH Advance diffractometer in reflection geometry, operating with a Kα anode (λ = 1.54178 Å) at 40 kV and 40 mA. Samples were ground and mounted as loose powders onto a Si sample holder. PXRD patterns were collected from 2 to 60 2θ-degrees with a step size of 0.02 degrees and an exposure time of 2 seconds per step.

Thermogravimetric analyses (TGA) were performed using a TGA/DSC 3^+ thermal analysis system under a N_2 atmosphere from room temperature to 800 °C at a ramping rate of 2 °C /min.

Attenuated total reflectance Fourier-transform infrared spectrometry (ATR-FTIR) was conducted using a Nicolet 6700 spectrometer (Thermo Scientific, USA), in the wavenumber range of 400-4000 cm^{-1} with a resolution of 4 cm^{-1}. The technique of attenuated total reflectance (ATR) was used for measurements.

Field-emission scanning electron microscopy (FESEM) was performed using a ZEISS GeminiSEM500. Cu_2O NCs, COF, and all xCu@COF NCs were observed directly without gold coating in nanoVP mode.

High-resolution transmission electron microscopy (HRTEM) images were obtained using a Talos F200S G2 EDS SUPER X system. Cu_2O NCs and all Cu_2O@x-COF NCs catalysts were prepared on a carbon grid after suspension in ethanol.

Electrode preparation: The electrodes for H-type cell electrochemical measurements were prepared on 1×2 cm^2 carbon paper (Alfa Aesar, Toray Carbon Paper, GGP-H-60) and the coverage area of the electrocatalyst was 1×1 cm^2. The stock solutions of as-prepared catalysts were drop-cast on each side of the carbon paper to yield 50 μg on the electrode for each catalyst. After that, the electrodes were dried for 30 minutes to ensure the complete evaporation of ethanol.

Electrochemical measurements: Electrocatalytic measurements were conducted in an H-cell using a Gamry Reference 600+ potentiostat/Galvano state/ZRA, equipped with a Nafion 117 ion-exchange membrane for separating the anodic and cathodic compartments. Furthermore, a leak-free Ag/AgCl reference electrode (LF-1, Alvatek) was placed near the working electrode in the cathodic compartment. A platinum gauze electrode (MaTecK, 3,600 mesh cm-2) assisted as the counter-electrode in the anodic compartment. 0.1 M $NaNO_3$ was used as catholyte, and 0.1 M Na_2SO_4 was used as an anolyte, purged with 99.99% Ar gas for 20 mins. The products were determined from the electrolyte after every 2 hours using a Molecular Devices SpectraMax ABS plus.

Determination of NH_3:

The concentration of generated NH_3 was quantified using the indophenol blue method. Each measurement was performed in triplicate to ensure reproducibility, and the resulting data were used to calculate error bars. After the NO_3^-RR test, 2 mL of the diluted electrolyte was collected for colorimetric analysis. To this solution, 2 mL of a reagent mixture containing 1 M NaOH, 5 wt% salicylic acid, and 5 wt% sodium citrate was added, followed by 0.2 mL of sodium nitroferricyanide solution (5 mg mL^{-1}) and 1 mL of NaClO solution (available chlorine 4.0 wt%). The mixture was then incubated for 2 h at room temperature to allow complete color development. The absorbance was recorded at 655 nm using a UV–Vis spectrophotometer.

Determination of NO_2^-:

Quantification of NO_2^- was conducted using a modified Griess colorimetric method. The colour reagent was prepared by dissolving p-aminobenzenesulfonamide (4 g) and N-(1-naphthyl)ethylenediamine dihydrochloride (0.2 g) in 50 mL of ultrapure water, followed by the addition of 10 mL of phosphoric acid (density 1.70 g mL^{-1}). The electrolyte sample (5 mL) was diluted appropriately to fall within the detection range, and 0.1 mL of the prepared color reagent was added. The solution was thoroughly mixed and allowed to stand for 30 min at room temperature for color development. Absorbance was measured at 540 nm using a UV–Vis spectrophotometer. Calibration curves were established using standard sodium nitrite solutions of known concentrations in the same electrolyte

Determination of N_2H_4:

The presence of hydrazine (N_2H_4) in the electrolyte was determined by the Watt-Chrisp method. A color reagent was prepared by mixing 100 mL of ethanol, 2 g of p-(dimethylamino)benzaldehyde, and 12 mL of concentrated HCl. Subsequently, 2 mL of this color reagent was added to 2 mL of the electrolyte. After 30 min of incubation at room temperature, the absorbance was recorded at 458 nm.

A calibration curve was generated using standard N_2H_4 solutions of known concentrations prepared in the same electrolyte.

Calculation of Faradaic Efficiency, Conversion Rate, Yield Rate, and Selectivity:

The Faradaic efficiency of was calculated using the following equation:

$$\text{Faradaic efficiency}_{NH_3} = (8F \times C \times V \times n)/Q$$

where F is the Faraday constant (96,485 C mol^{-1}), C is the measured concentration of NH_3 (mol mL^{-1}), V is the electrolyte volume (mL), n is the dilution factor, and Q is the total charge passed during electrolysis (C).

The nitrate conversion rate was determined according to the following equation:

$$\text{Conversion} = \Delta c_{NO_3^-}/c_0 \times 100\%$$

Where $\Delta c_{NO_3^-}$ represents the decrease in nitrate concentration before and after electrolysis (mol cm^{-3}), and c_0 denotes the initial nitrate concentration (mol cm^{-3})

The yield rate was calculated using the following equation:

$$\text{Yield rate} = (C \times V \times n)/(t \times A)$$

where C is the concentration of NH_3 (mol mL^{-1}), V is the volume of electrolyte (mL), n is the dilution factor, t is the electrolysis time (h), and A is the geometric area of the working electrode (cm^2).

The selectivity of ammonia or nitrite was calculated according to the following equation

$$\text{Selectivity} = c/\Delta c_{NO_3^-} \times 100\%$$

where c represents the concentration of the generated product in aqueous solution, $\Delta c_{NO_3^-}$ is the variation in nitrate concentration before and after electrolysis (mol L^{-1}).

Each reported data point was measured at least three times and is therefore presented with average values.

Isotopic ^{15}N-labeling experiment:

For isotopic labelling studies, a 0.1 M $Na^{15}NO_3$ and 0.1 M Na_2SO_4 solution was used as the electrolyte in the cathode compartment. Subsequently, 50 µL of deuterium oxide (D_2O) was introduced to 0.5 mL of the electrolyte, and a certain amount of hydrochloric acid was added for acidification to facilitate the ^{1}H NMR (400 MHz) detection. For comparison, NH_3 detection via NMR was also performed using an electrolyte containing 0.1 M $Na^{14}SO_4$ and 1 M $Na^{14}NO_3$, following the same procedure.

Molecular dynamics simulation: As illustrated in Figure 6 of the manuscript, a three-layer COF membrane with periodic boundaries in the X–Y plane was used as the model. The simulation box was initially filled with water molecules, with two COF membranes dividing the system into three regions along the Z direction. The top and bottom regions had approximately equal volumes to the central area (about 3 nm in the Z direction). The top and bottom regions were first filled with 44 Na^+ and 44 NO_3^-, with the middle region filled with 44 NH_3 molecules. In our molecular dynamics (MD) simulations, the $NaNO_3$ concentration was set to approximately 0.5 M, which is slightly higher than the experimental condition (0.1 M). This adjustment is a common practice in atomistic simulations to accelerate ion dynamics and achieve statistically meaningful sampling within feasible computational timescales. While the absolute concentration is higher, this does not alter the relative ion-COF interaction trends or transport pathways that govern the mechanistic interpretation. The observed preferential ion distribution and coordination behavior therefore remain representative of the experimental system. The periodic boundary conditions were applied to all three directions. To prevent the drift of the COF frameworks, the N atoms of TBA monomers were fixed while the rest of the atoms were flexible. Nonbonding interactions were adopted by $\sum 4\varepsilon_{ij}\left[\left(\frac{\sigma_{ij}}{r_{ij}}\right)^{12}-\left(\frac{\sigma_{ij}}{r_{ij}}\right)^{6}\right]+\sum\frac{q_i q_j}{4\pi\varepsilon_0 r_{ij}}$, where ε_{ij} and σ_{ij} are the well and collision diameter of the Lennard-Jones (LJ) potential, r_{ij} is the distance between atom i and j, q_i is the atomic charge of atom i, and ε_0 is the permittivity of vacuum. The LJ potential parameters were adopted from the universal force field (UFF)[1] with the help of the OBGMX tool.[2] The atomic charges of the COFs were generated using DDEC methods.[3] Water was modeled by the SPC/E model,[4] and the NH_3 molecules were described by the OPLS-AA force field.[5] Force field for Na^+ and NO_3^- were adopted from literature.[6] The electrostatic interactions were calculated with the Particle-Mesh Ewald method, while the LJ interactions were calculated using a cutoff of 1.2 nm. The temperature was maintained at 300 K using a v-rescale scheme, and the time step was 1 fs. After equilibration, a constant number of particles, volume, and temperature (NVT) ensemble of 20 ns was used for output simulation. All the MD simulations were performed using GROMACS 2021,[7] and the model was visualized by VMD 1.9.[8]

Section 2. Synthesis of Cu_2O, COF, and Cu_2O@x-COF

Synthesis of Cu_2O nanocubes (NCs): Cu_2O NCs were fabricated using the wet chemical reduction method, as reported previously. [9] In a typical synthesis, anhydrous $CuCl_2$ (0.171 g) was dissolved in 100 mL of water. The solution was then stirred for 15 minutes. After that, 10 mL NaOH aqueous solution (2.0 M) was then added slowly drop-wise into the above light green solution. The solution changed to a brown color. After vigorous magnetic stirring for 30 minutes, 10 mL of an ascorbic acid

solution (0.6 M) was added to the above solution. The color of the liquid gradually changed from blue to a turbid red. The mixture was aged for 3 h. All procedures were carried out at a temperature of 55 °C under constant vigorous magnetic stirring. The resulting precipitate was collected from the solution by centrifugation at 6,000 r.p.m. for 5 min and washed several times with deionized water and ethanol. The resulting Cu_2O was then dried under vacuum for 12 hours.

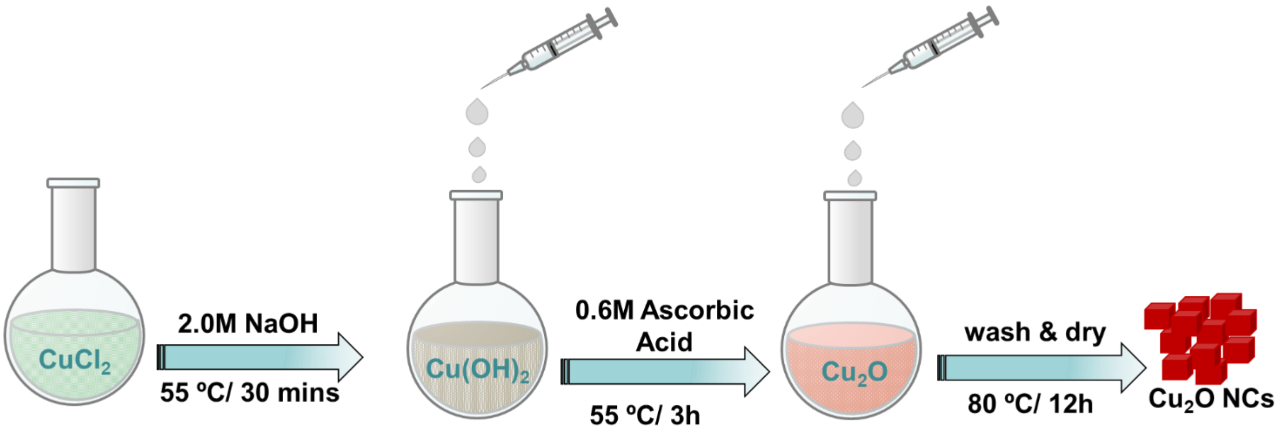

Scheme 1. A schematic diagram of the Cu_2O NCs preparation.

Synthesis of Py-COF: A Pyrex glass Schlenk tube (15 mL) was charged with 2,4,6-tris(4-aminophenyl) triazine (60 mg, 0.15 mmol), 4,4',4''-trinitrilotribenzaldehyde (56 mg,0.15 mmol), 2-vinylpyridine (60 µL, 0.6 mmol), BF_3•OEt_2 (10 µL, 0.1 mmol), and acetic acid (100 µL, 6M) in o-dichlorobenzene (o-DCB)/n-BuOH (2 / 2 mL). The mixture was first sonicated for 0.5 h to form a bulk solid, and then a Schlenk tube was flashed frozen at 77 K (in a liquid N_2 bath) and degassed by three freeze-pump-thaw cycles. The internal pressure was evacuated to 10^{-3} mbar. Afterwards, the tube was sealed and heated at 120 °C for 3 days. The bright yellow precipitate was washed with acetone and tetrahydrofuran several times and collected by filtration. Finally, the powder was dried in a normal oven at 80 °C. Yield = 89.7 % (93 mg). Anal. Calcd. (%): C, 81.10; H, 3.89; N, 15.01. Found (%): C, 78.85; H, 5.10; N, 16.05.

Synthesis of Im-COF: A Pyrex glass Schlenk tube (15 mL) was charged with 2,4,6-Tris(4-aminophenyl) triazine (60 mg, 0.15 mmol), 4,4',4''-trinitrilotribenzaldehyde (56 mg,0.15 mmol), 1-vinylimidazole (60 µL, 0.6 mmol), BF_3•OEt_2 (10 µL, 0.1 mmol), and acetic acid (100 µL, 6M) in o-dichlorobenzene (o-DCB)/n-BuOH (2 / 2 mL). The mixture was first sonicated for 0.5 h to form a bulk solid, and then a Schlenk tube was flashed frozen at 77 K (in a liquid N_2 bath) and degassed by three freeze-pump-thaw cycles. The internal pressure was evacuated to 10^{-3} mbar. Afterwards, the tube was sealed and heated at 120 °C for 3 days. The bright yellow precipitate was washed with acetone and tetrahydrofuran several times and collected by filtration. Finally, the powder was dried in a normal

oven at 80 °C. Yield = 89.7 % (93 mg). Anal. Calcd. (%): C, 81.10; H, 3.89; N, 15.01. Found (%): C, 78.85; H, 5.10; N, 16.05.

Synthesis of core-shell Cu_2O@x-COFs**:** Core-shell Cu_2O@x-COF NCs were simply synthesized by a wet-chemical process in a Pyrex glass Schlenk tube. COF organic precursors with different ratios of Cu_2O cubes added to the Schlenk tubes. 3 mL of n-BuOH and 3mL of o-dichlorobenzene with 2-vinylpyridine (60 µL, 0.6 mmol), $BF_3.OEt_2$ (10 µL, 0.1 mmol) and acetic acid (100 µL, 6M) were also added to the above Schlenk tubes as a solvent. The mixture was sonicated for 30 minutes until a homogenous solution was obtained. The Schlenk tube underwent a degassing and evacuation process three times using freeze-pump-thaw cycles, with flashing frozen at 77 K (in a liquid N_2 bath). After that, the Schlenk tube with an internal pressure of 10^{-3} mbar was sealed and heated to 120 ºC for 3 days. The red precipitates were washed with a solvent mixture (Methanol, Dimethyl Ether, and Acetone) several times. Finally, the red powder was collected after Soxhlet extraction for 12 h and dried at 80 ºC for 6 h.

Supplementary Figures:

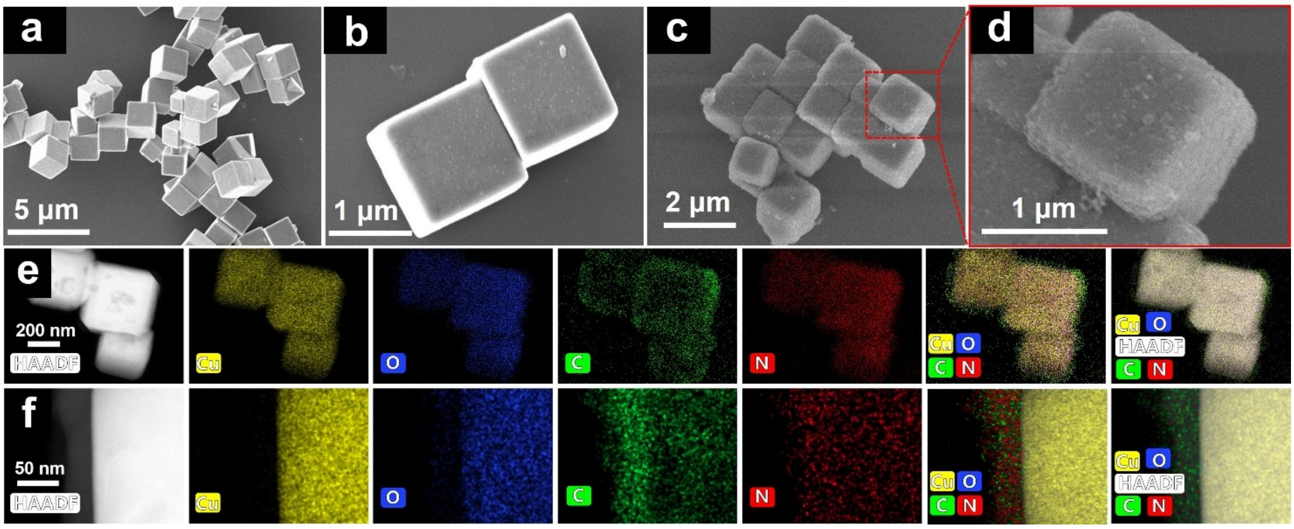

Supplementary Fig. 1. SEM images of (a-b) pristine Cu_2O, (c-d) Cu_2O@Py-COF. HRTEM images, and (e-f) HAADF-STEM EDS mapping images Cu_2O@Py-COF.

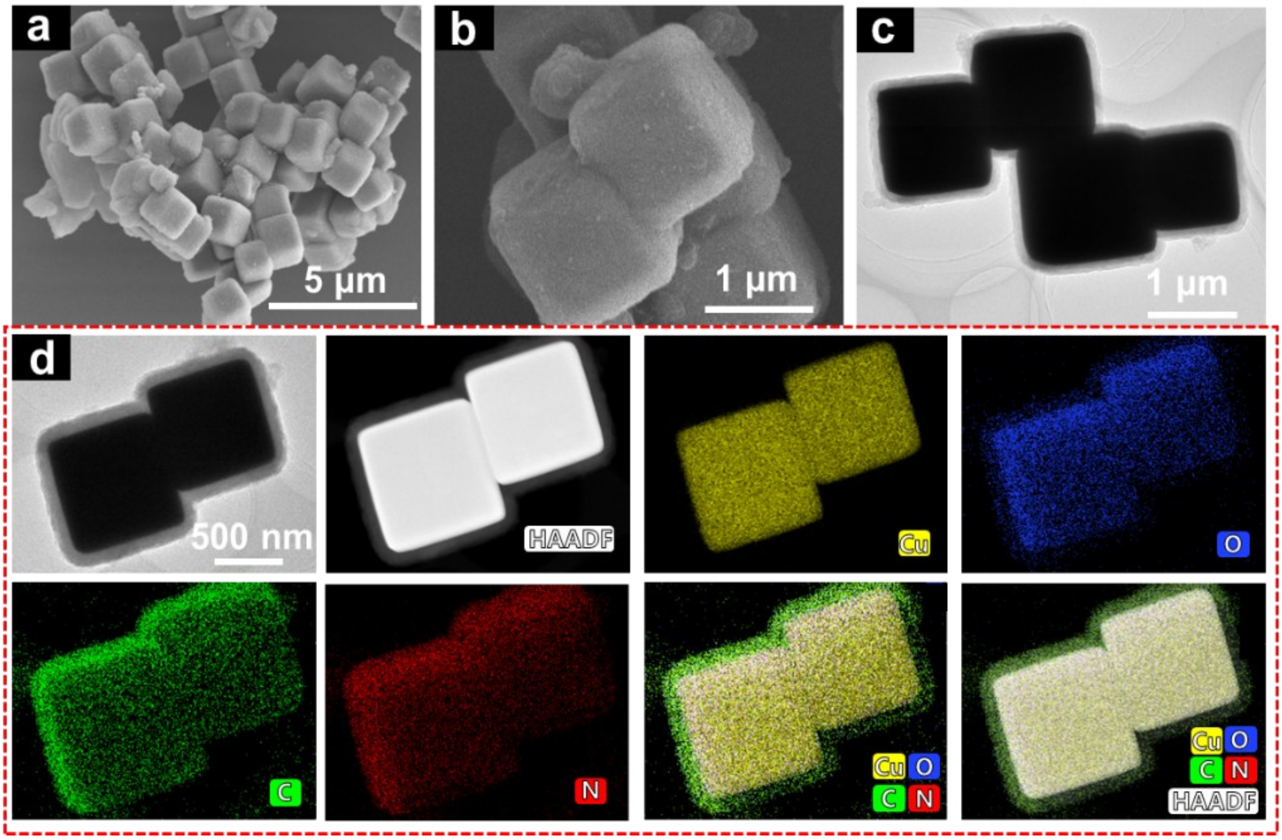

Supplementary Fig. 2. (a-b) SEM, (c) HRTEM images, and (d) HAADF-STEM EDS mapping images Cu_2O@Im-COF.

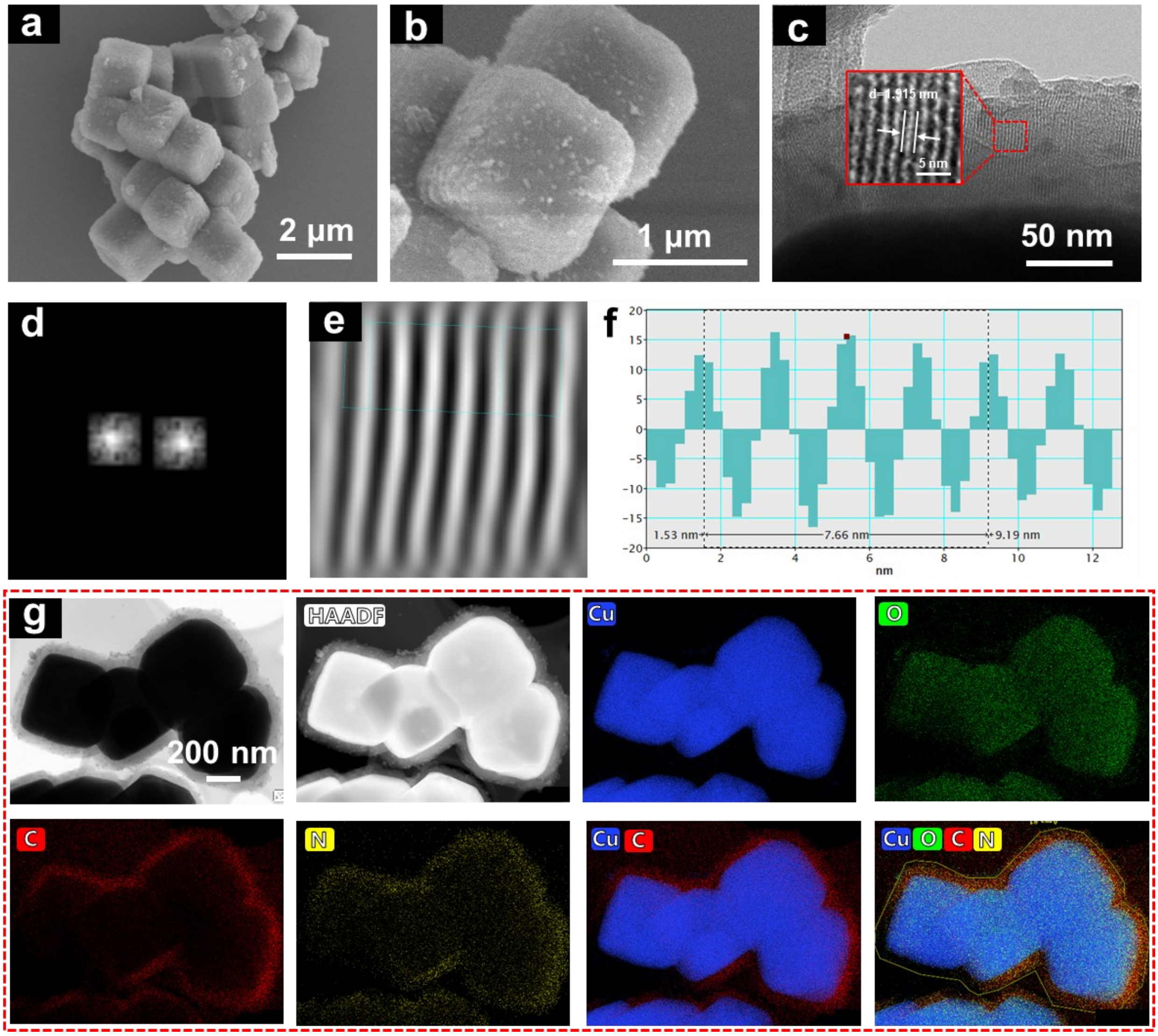

Supplementary Fig. 3. (a-b) SEM, (c) HRTEM images, (d) FFT patterns (e) iFFT, (f) line intensity profile and (g) HAADF-STEM EDS mapping images Cu_2O@Py-COF-75.

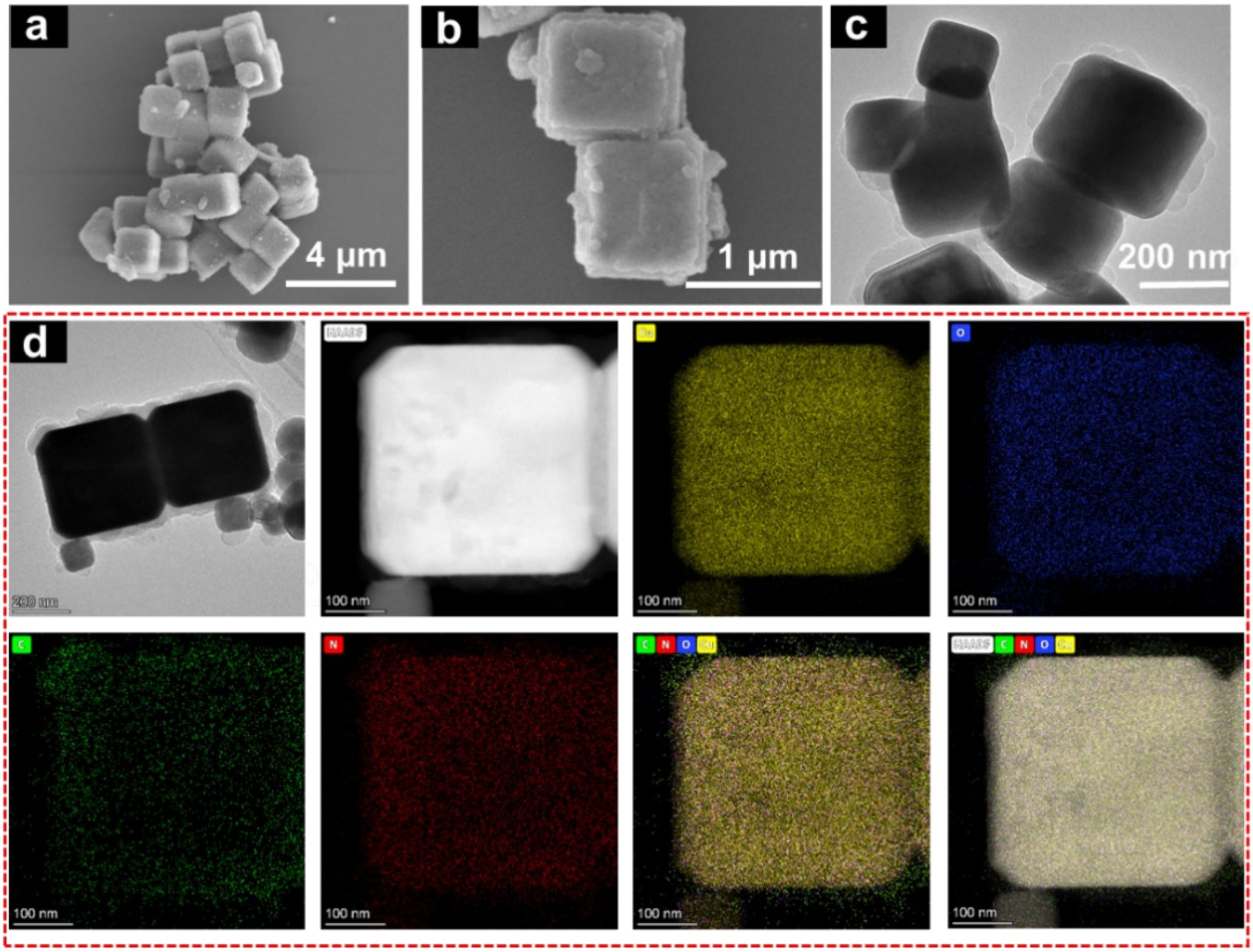

Supplementary Fig. 4. (a-b) SEM, (c) HRTEM images, and (d) HAADF-STEM EDS mapping images Cu_2O@Py-COF-25.

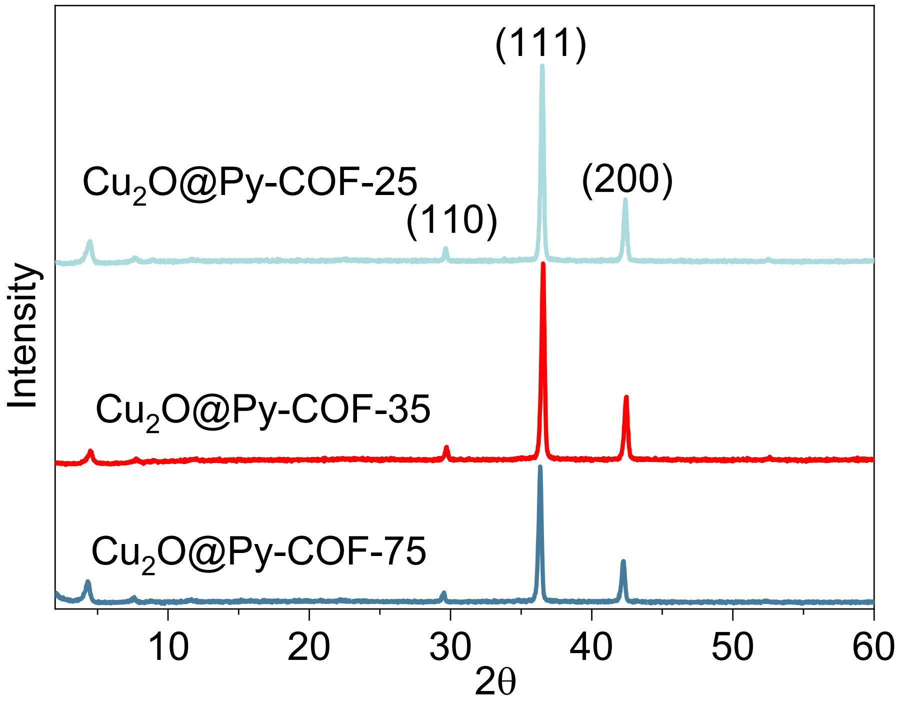

Supplementary Fig. 5. PXRD Patterns of Cu_2O@ Py-COF-75, Cu_2O@ Py-COF-35, and Cu_2O@ Py-COF-25.

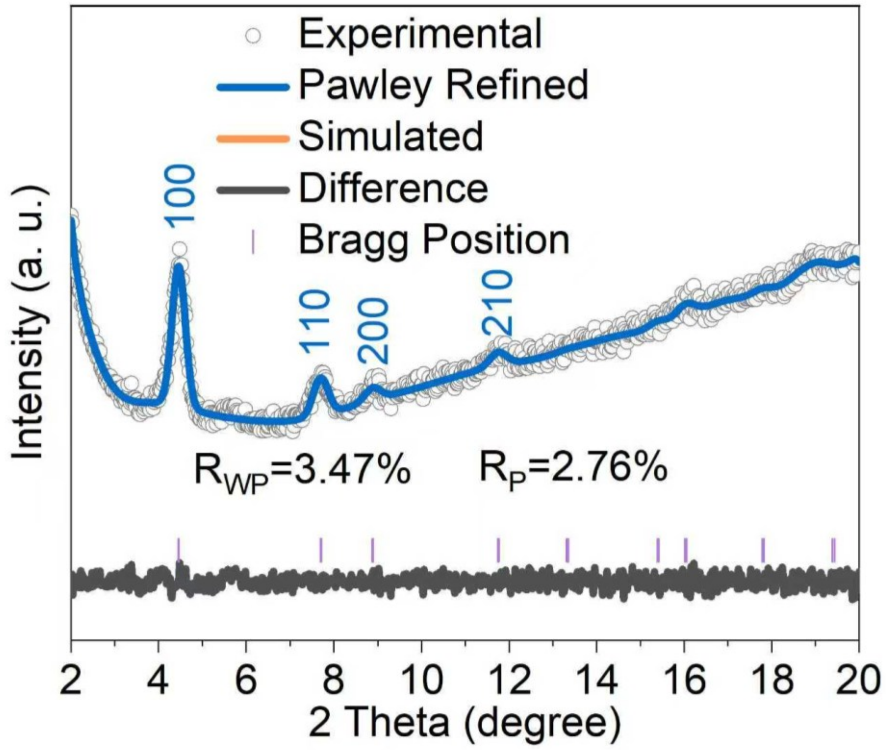

Supplementary Fig.6. The corresponding PXRD patterns for Im-COF: experimental (grey circle line), Pawley refined (blue line), simulated pattern for AA stacking (yellow line), and Bragg positions (purple line).

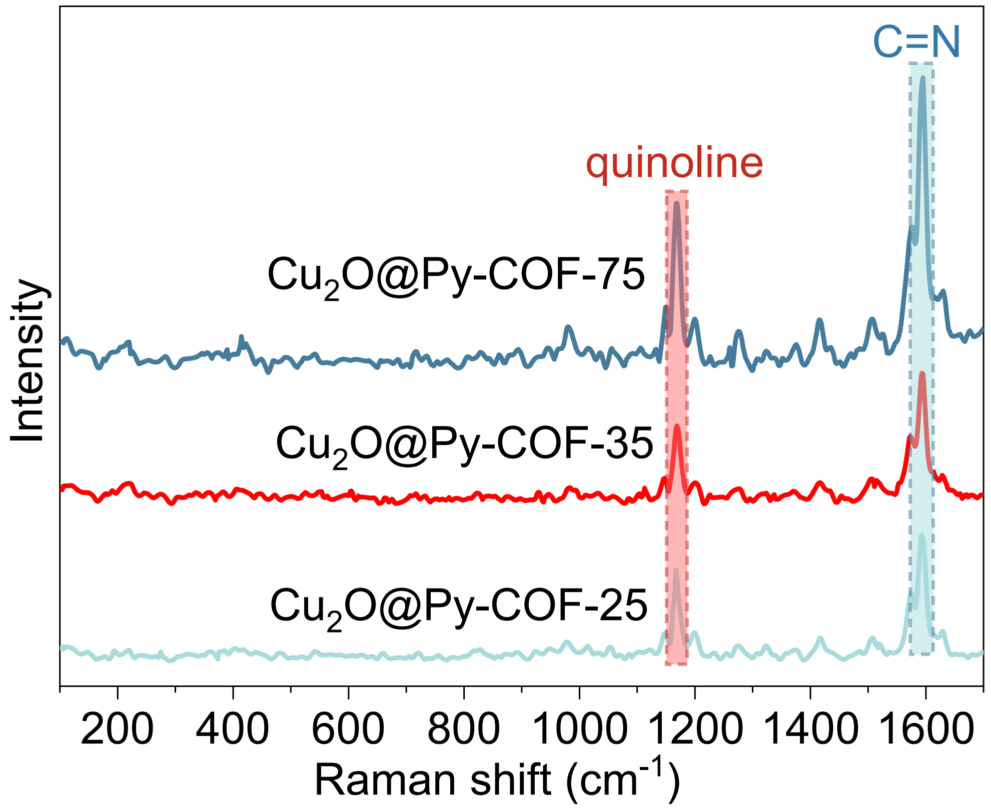

Supplementary Fig. 7. Raman spectra of Cu_2O@Py-COF-75, Cu_2O@ Py-COF-35, and Cu_2O@ Py-COF-25.

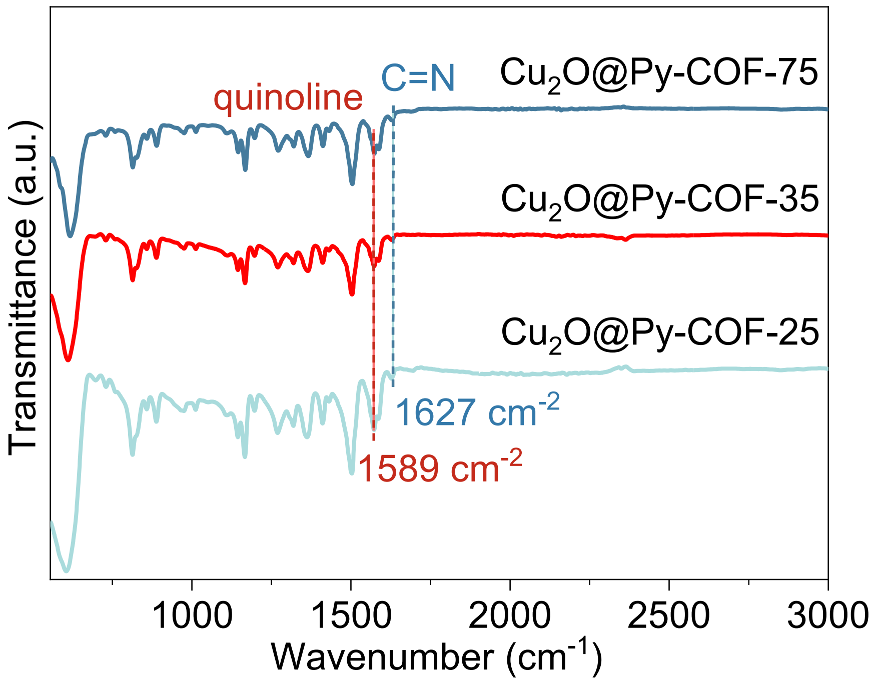

Supplementary Fig. 8. FTIR spectra of Cu_2O@Py-COF-75, Cu_2O@Py-COF-35, and Cu_2O@Py-COF-25.

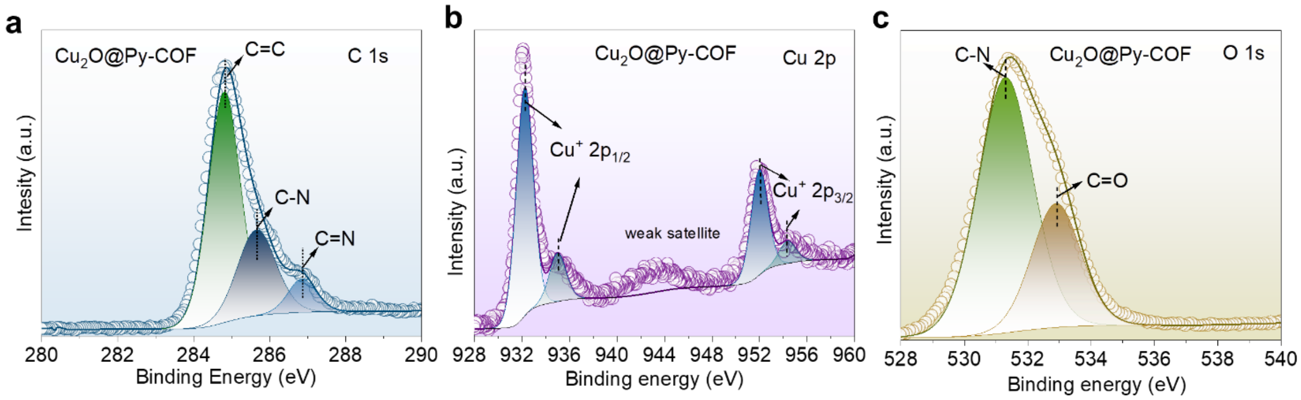

Supplementary Fig. 9. XPS spectra (a) C 1s, (b) Cu 2p, and (c) O 1s of Cu_2O@Py-COF electrocatalyst.

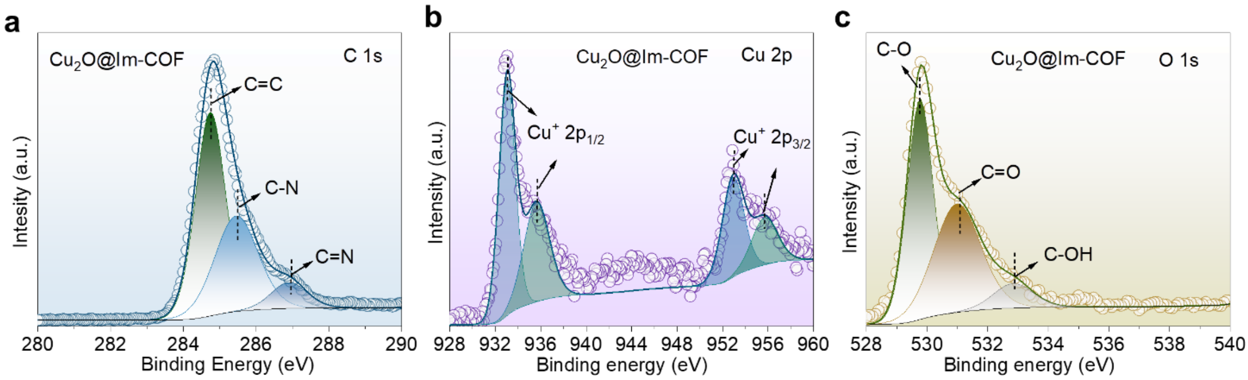

Supplementary Fig. 10. XPS spectra (a) C 1s, (b) Cu 2p, (c) O 1s of Cu_2O@Im-COF electrocatalyst.

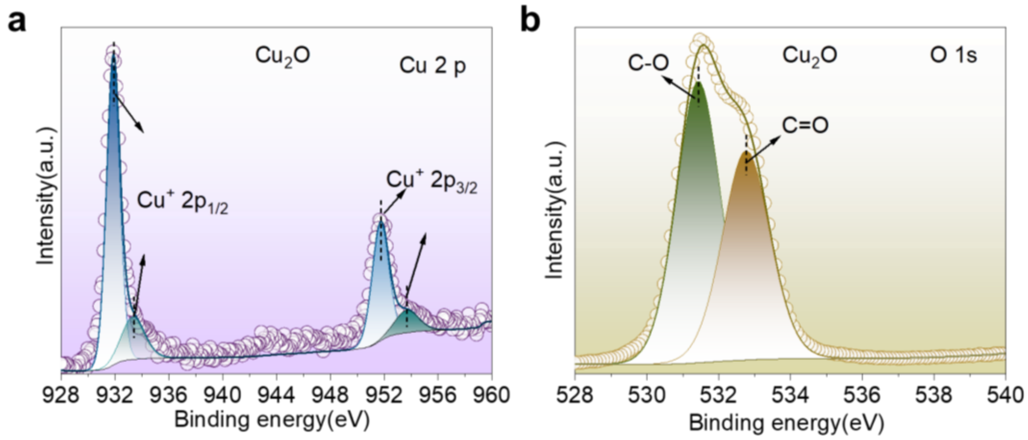

Supplementary Fig. 11. XPS spectra (a) Cu 2p and (b) O1s of Cu_2O NCs.

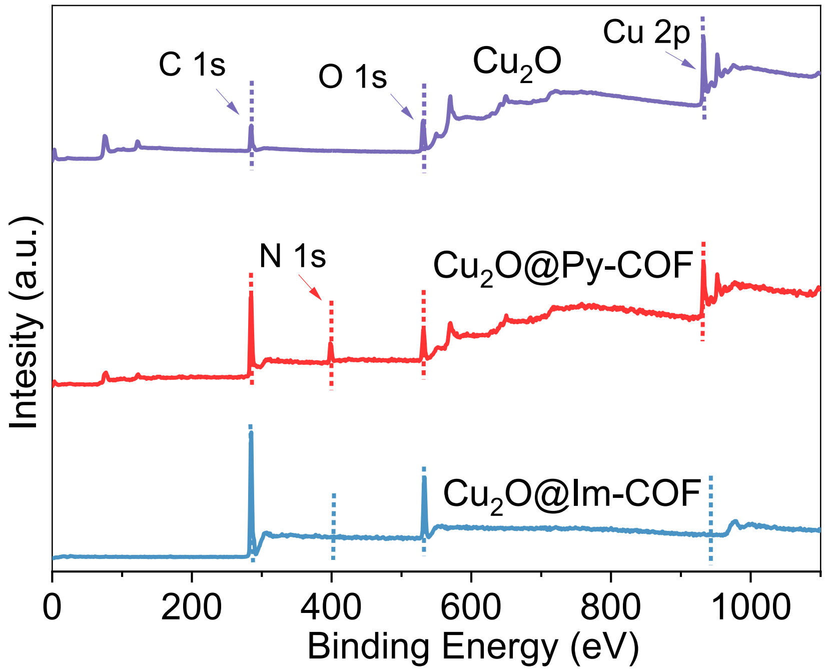

Supplementary Fig. 12. XPS survey spectra of Cu_2O NCs, Cu_2O@Py-COF and Cu_2O@Im-COF.

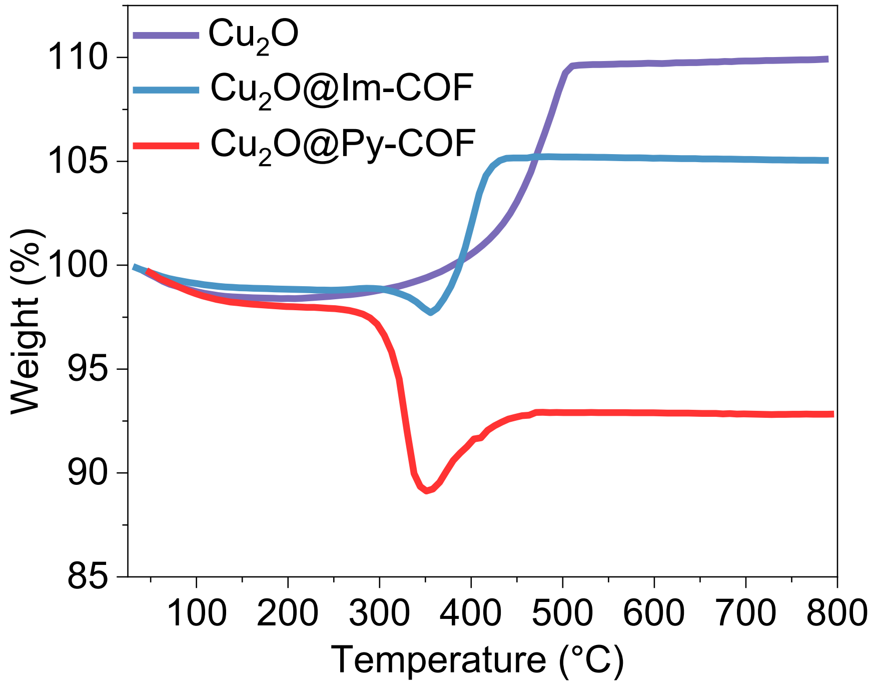

Supplementary Fig. 13. Thermogravimetric analysis (TGA) of Cu_2O NCs, Cu_2O@Py-COF and Cu_2O@Im-COF.

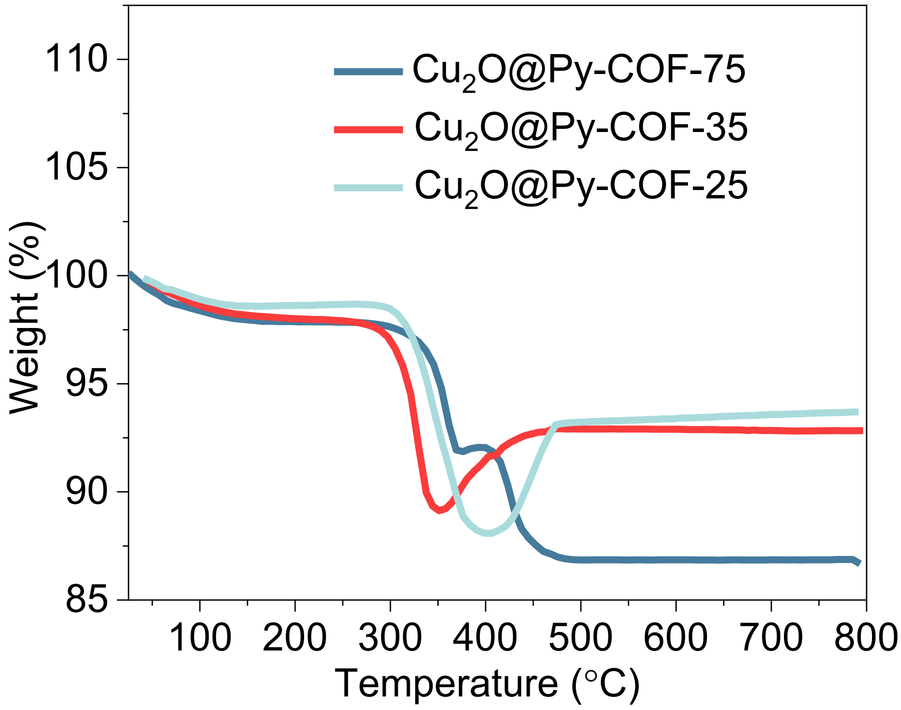

Supplementary Fig. 14. TGA curves of Cu_2O@Py-COF-75, Cu_2O@Py-COF-35, and Cu_2O@Py-COF-25.

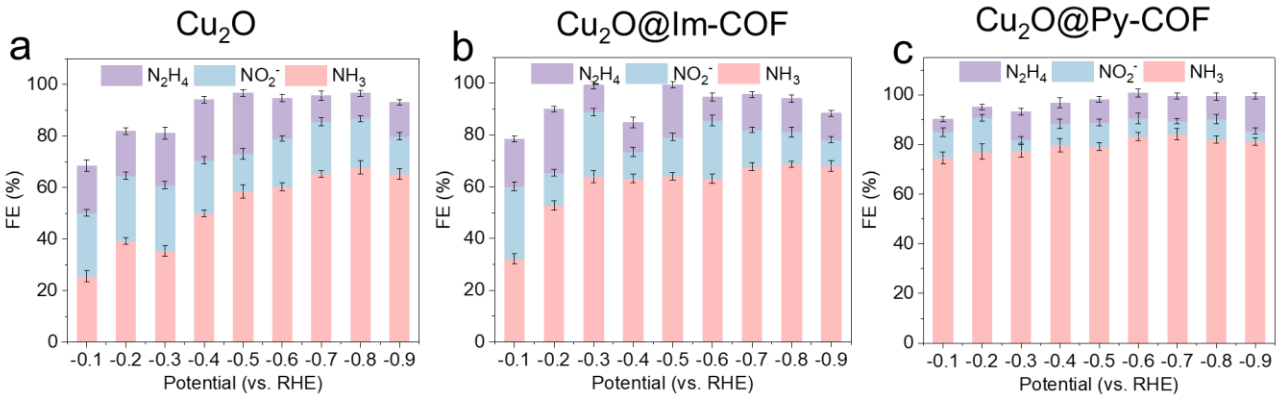

Supplementary Fig. 15. Quantitative analysis of by-products (NH_3, NO_2^-, and N_2H_4) during electrochemical nitrate reduction over (a) Cu_2O, (b) Cu_2O@Im-COF, and (c) Cu_2O@Py-COF catalysts at various applied potentials.

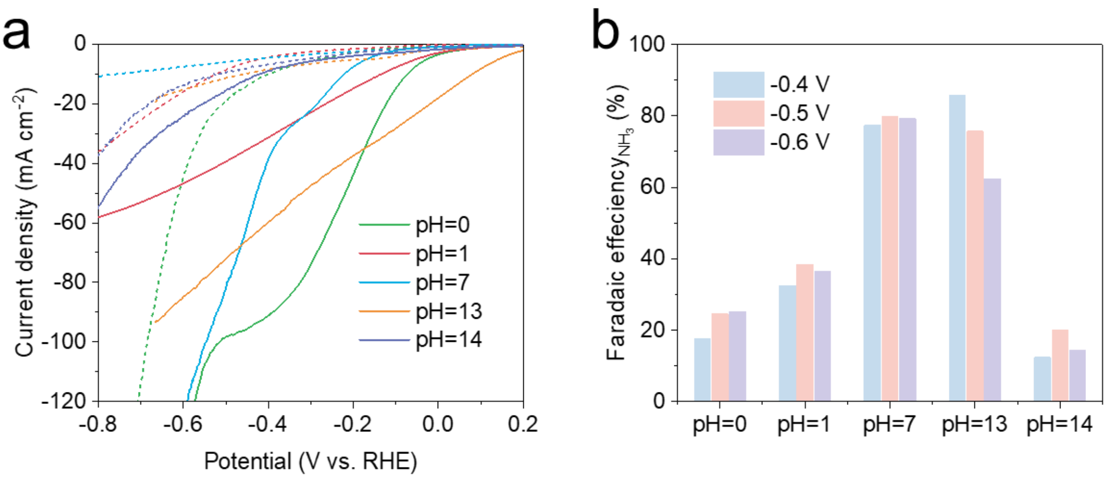

Supplementary Fig. 16. (a) Linear sweep voltammetry (LSV) curves of Cu_2O@Py-COF recorded in electrolytes of varying pH (0, 1, 7, 13, and 14), and (b) corresponding Faradaic efficiency (FE) values for NH_3 production measured at three different potentials (-0.5, -0.7, and -0.9 V vs. RHE) under each pH condition.

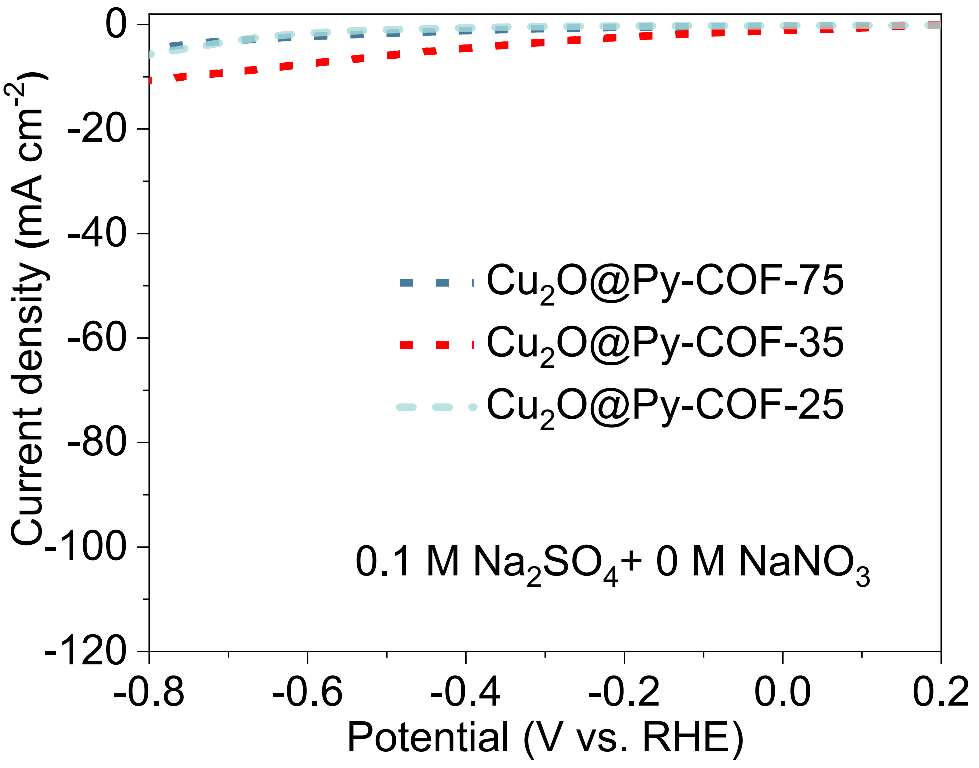

Supplementary Fig. 17. Linear sweep voltammetry (LSV) curves in Ar-saturated 0.1 M Na_2SO_4 without $NaNO_3$ over the Cu_2O@Py-COF-75, Cu_2O@Py-COF-35, and Cu_2O@Py-COF-25 electrocatalysts.

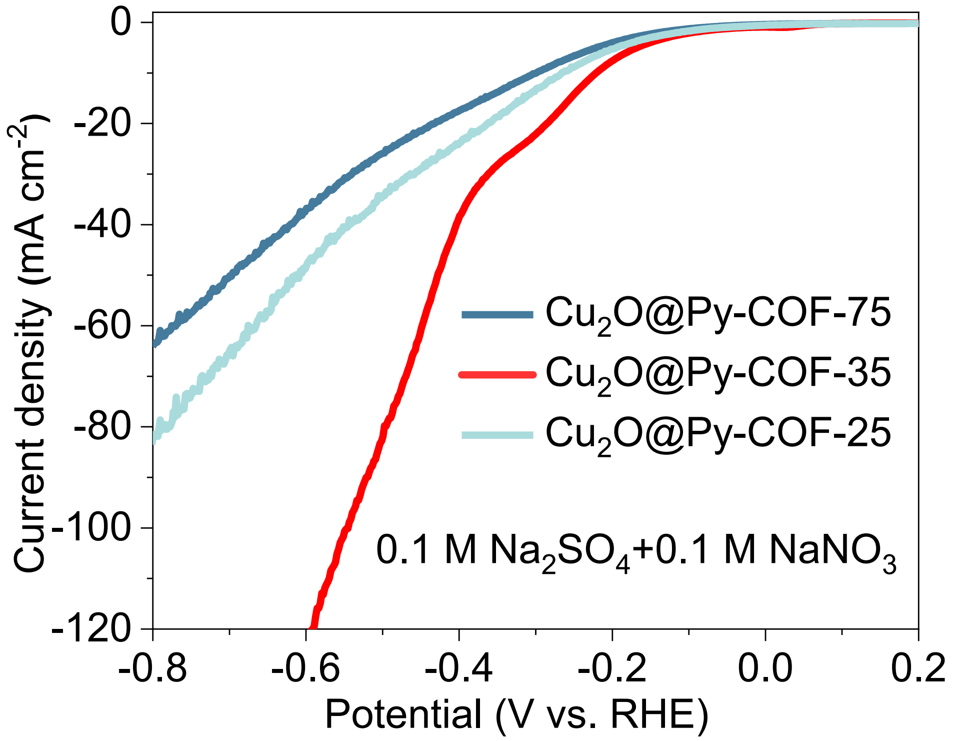

Supplementary Fig. 18. Linear sweep voltammetry (LSV) curves in Ar-saturated 0.1M Na_2SO_4 with $NaNO_3$ over the Cu_2O@Py-COF-75, Cu_2O@Py-COF-35, and Cu_2O@Py-COF-25 electrocatalysts.

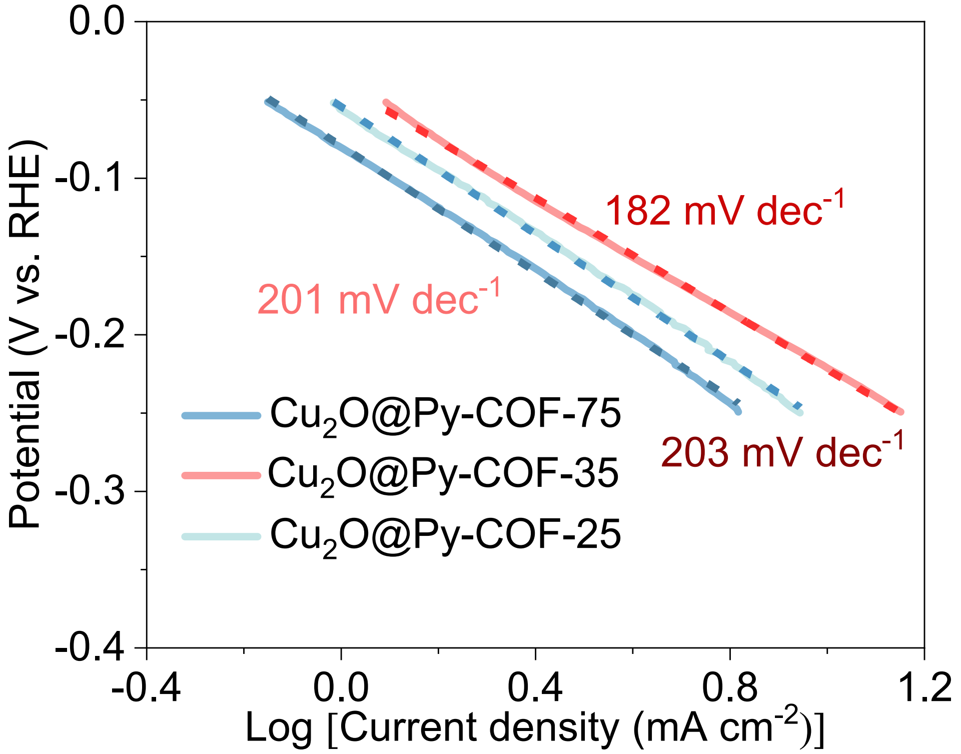

Supplementary Fig. 19. Tafel plots at different potentials over the Cu_2O@Py-COF-75, Cu_2O@Py-COF-35, and Cu_2O@Py-COF-25 electrocatalysts.

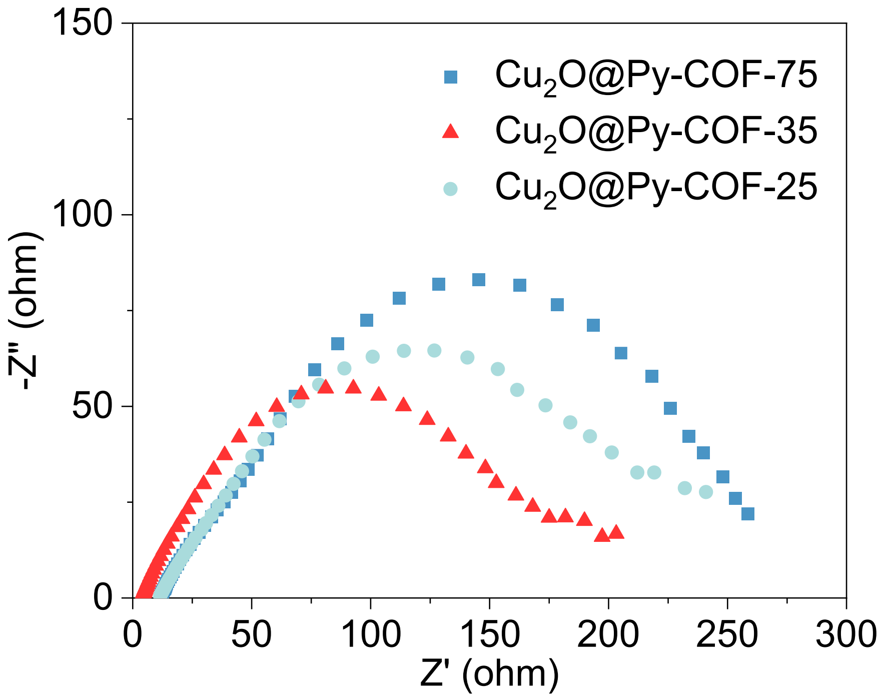

Supplementary Fig. 20. EIS Nyquist plot of the Cu_2O@Py-COF-75, Cu_2O@Py-COF-35, and Cu_2O@Py-COF-25 electrocatalysts.

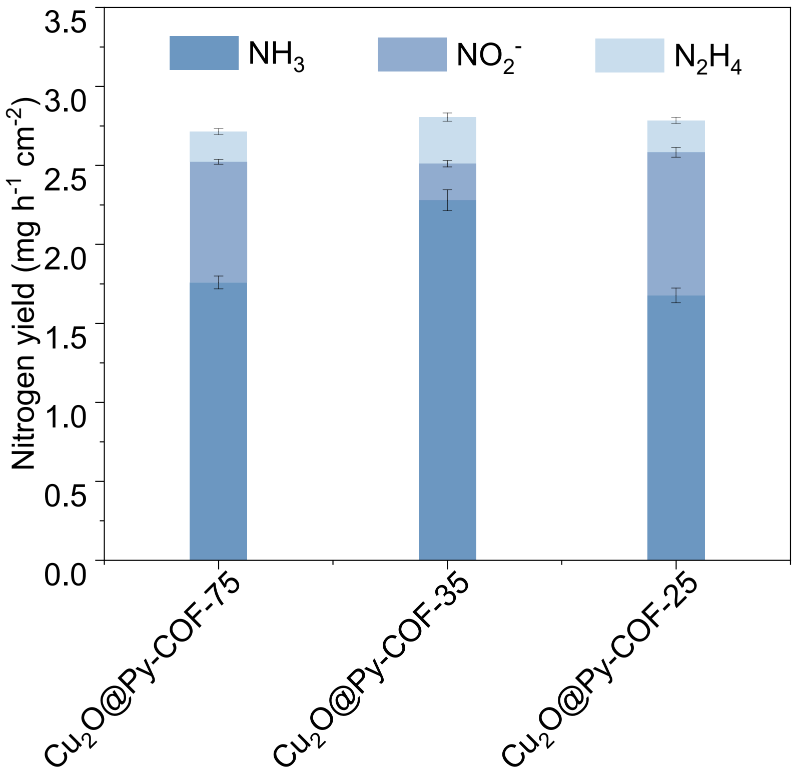

Supplementary Fig. 21. The comparisons of NH_3 yield rate (histogram) over the Cu_2O@Py-COF-75, Cu_2O@Py-COF-35, and Cu_2O@Py-COF-25 electrocatalysts.

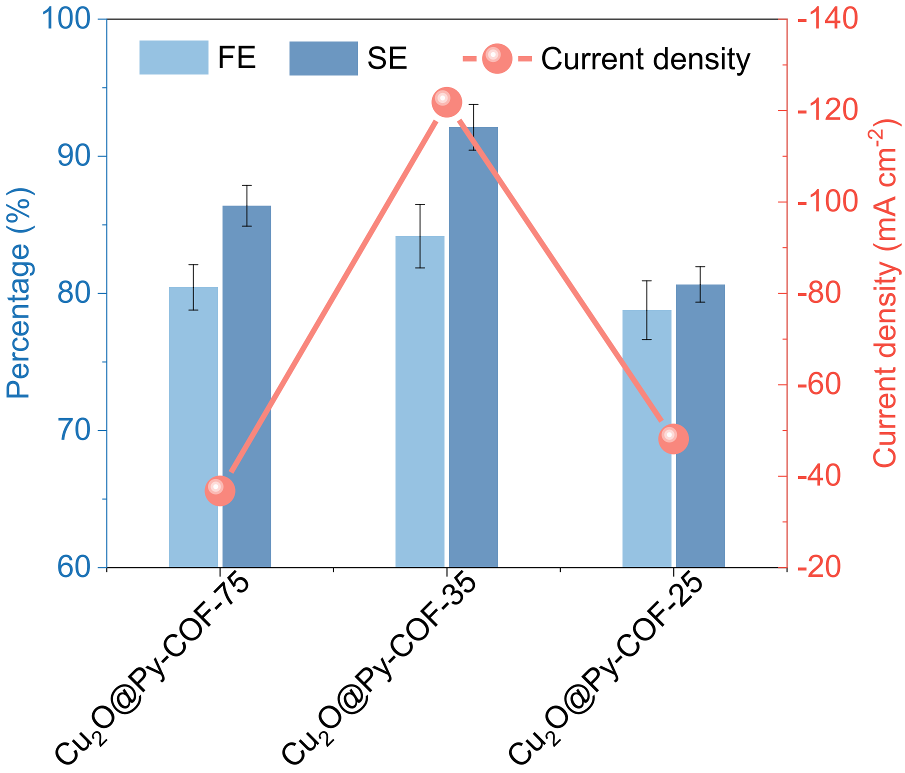

Supplementary Fig. 22. Potential-dependent FE_{NH3}, NH_3 selectivity, and current densities over the Cu_2O@Py-COF-75, Cu_2O@Py-COF-35, and Cu_2O@Py-COF-25 electrocatalysts.

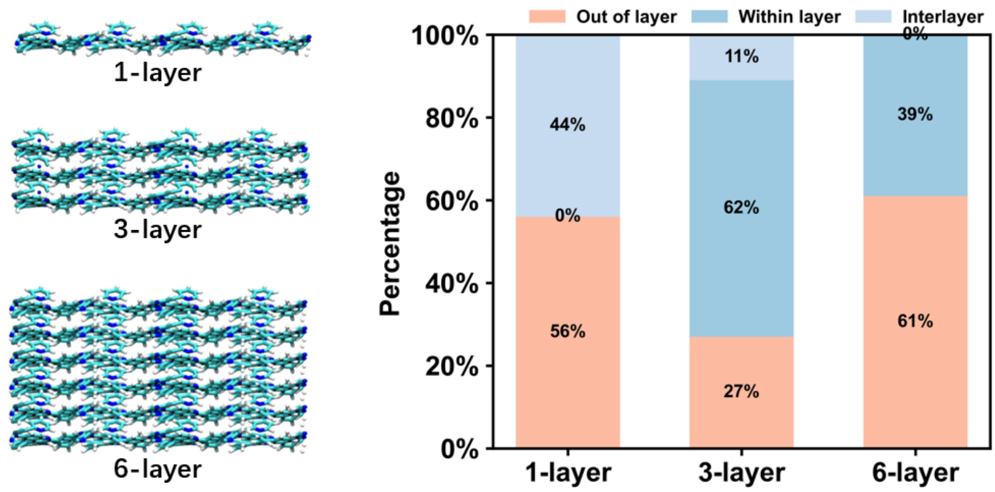

Supplementary Fig. 23. Percentage of NO_3^- in different regions after 20 ns with different thicknesses (1-, 3-, and 6-layer models).

Table 1. Electrochemical performance comparison of core-shell Cu_2O@x-COF materials with recently reported electrocatalysts for nitrate reduction to ammonia.

**Electrocatalyst**	**Electrolyte**	**Potential**	**FE_{NH3} (%)**	**NH_3 rate**	**Ref.**
**Cu_2O@Py-COF**	0.1 M Na_2SO_4 +0.1 M $NaNO_3$	−0.7 V vs. RHE	84	2.27 mg h^{-1} cm^{-2}	This work
**Cu_2O@Im-COF**			69	0.84 mg h^{-1} cm^{-2}	
**Cu_2O NCs**			65	0.64 mg h^{-1} cm^{-2}	
**Cu(111) nanodisks**	0.1 M KOH+10 mM KNO_3	−0.63 V vs. RHE	81.11	2.16 mg h^{-1} cm^{-2}	[10]
**1-Cu**	0.5 M Na_2SO_4+5 mM $NaNO_3$	−0.9 V vs. RHE	85.5	1.12 mg h^{-1} cm^{-2}	[11]
**Cu_2O**	0.5 M Na_2SO_4 +200 ppm NO_3^-	−0.58 V vs. RHE	60	0.595 mg h^{-1} cm^{-2}	[12]
**Cu incorporated PTCDA**	0.1 M PBS+0.1 M NO_3^-	−0.4 V vs. RHE	77	0.442 mg h^{-1} cm^{-2}	[13]
**Cu nanotubes**	0.5 M K_2SO_4+50 mg $L^{-1}NO_3^-$–N	−0.65V vs. RHE	85.70	0.78 mg h^{-1} cm^{-2}	[14]
**CuPd aerogels**	0.5 M K_2SO_4+50 mg $L^{-1}NO_3^-$–N	−0.46 V vs. RHE	90.02	0.78 mg h^{-1} cm^{-2}	[15]
**Cu@Cu_{2+1}O**	0.5 M K2SO4 3.6 mM NO3-	−1.2 V vs. SCE	87.1	0.58 mg h^{-1} cm^{-2}	[16]
**CuCl/TiO_2**	0.5 M Na_2SO_4+ 100 mg L^{-1} NO_3^-	−1.0 vs. RHE	95.6	2.142 mg h^{-1} cm^{-2}	[17]

**Cu_3N**	0.1 M Na_2SO_4 +0.1 M KNO_3	−0.6 vs. RHE	93.1	2.9 mg h^{-1} cm^{-2}	18
**L-Cu/Cu_2O**	1 M KOH + 0.1 M KNO_3	−0.2 V vs. RHE	86	2.55 mg h^{-1} cm^{-2}	19
**M- Cu/Cu_2O**			95	3.4 mg h^{-1} cm^{-2}	
**S- Cu/Cu_2O**			80	3.91 mg h^{-1} cm^{-2}	

Note: All NH_3 yield rates have been converted to units of mg h^{-1} cm^{-2} for consistency. Reported values originally given in μmol h^{-1} cm^{-2} were converted using the molar mass of NH_3 (17.03 g mol^{-1}), and values expressed in mg h^{-1} mg^{-1} of catalyst were normalized by the corresponding catalyst loading on the electrode surface area (typically mg cm^{-2}). The conversion follows:

Yield (mg h^{-1} cm^{-2}) =Yield (μmol h^{-1} cm^{-2}) ×17.03×10^{-3}

Or

Yield (mg h^{-1} cm^{-2}) =Yield (mg h^{-1} cm^{-2}) ×Loading (mg cm^{-2})

References

1. Rappé, A. K.; Casewit, C. J.; Colwell, K.; Goddard III, W. A.; Skiff, W. M., UFF, a full periodic table force field for molecular mechanics and molecular dynamics simulations. *J. Amer. Chem. Soc.* **1992,** *114*, 10024-10035.
2. Garberoglio, G., OBGMX: A web-based generator of GROMACS topologies for molecular and periodic systems using the universal force field. *J. Comp. Chem.* **2012,** 33, 2204-2208.
3. Manz, T. A.; Limas, N. G., Introducing DDEC6 atomic population analysis: part 1. Charge partitioning theory and methodology. *RSC Advances* **2016,** 6 (53), 47771-47801.
4. Berendsen, H. J.; Grigera, J.-R.; Straatsma, T. P., The missing term in effective pair potentials. *J. Phys. Chem.* **1987,** 91 (24), 6269-6271.
5. Jorgensen, W. L.; Maxwell, D. S.; Tirado-Rives, J., Development and testing of the OPLS all-atom force field on conformational energetics and properties of organic liquids. J *J. Amer. Chem. Soc.* **1996,** 118 (45), 11225-11236.
6. Joung, I. S.; Cheatham III, T. E., Determination of alkali and halide monovalent ion parameters for use in explicitly solvated biomolecular simulations. *J. Phys. Chem. B* **2008,** 112 (30), 9020-9041.
7. Abraham, M. J.; Murtola, T.; Schulz, R.; Páll, S.; Smith, J. C.; Hess, B.; Lindahl, E., GROMACS: High performance molecular simulations through multi-level parallelism from laptops to supercomputers. SoftwareX **2015,** 1, 19-25.
8. Humphrey, W.; Dalke, A.; Schulten, K., VMD: visual molecular dynamics. *Journal of Molecular Graphics* **1996,** 14 (1), 33-38.
9. Wu, Y. A.; McNulty, I.; Liu, C.; Lau, K. C.; Liu, Q.; Paulikas, A. P.; Sun, C.-J.; Cai, Z.; Guest, J. R.; Ren, Y., Facet-dependent active sites of a single Cu_2O particle photocatalyst for CO_2 reduction to methanol. *Nature Energy* **2019,** 4 (11), 957-968.
10. Wu, K.; Sun, C.; Wang, Z.; Song, Q.; Bai, X.; Yu, X.; Li, Q.; Wang, Z.; Zhang, H.; Zhang, J., Surface reconstruction on uniform Cu nanodisks boosted electrochemical nitrate reduction to ammonia. *ACS Materials Letters* **2022,** 4 (4), 650-656.
11. Xu, Y.-T.; Xie, M.-Y.; Zhong, H.; Cao, Y., In situ clustering of single-atom copper precatalysts in a metal-organic framework for efficient electrocatalytic nitrate-to-ammonia reduction. *ACS Catalysis* **2022,** 12 (14), 8698-8706.
12. Gong, Z.; Zhong, W.; He, Z.; Liu, Q.; Chen, H.; Zhou, D.; Zhang, N.; Kang, X.; Chen, Y., Regulating surface oxygen species on copper (I) oxides via plasma treatment for effective reduction of nitrate to ammonia. *Applied Catalysis B: Environmental* **2022,** 305, 121021.
13. Chen, G.-F.; Yuan, Y.; Jiang, H.; Ren, S.-Y.; Ding, L.-X.; Ma, L.; Wu, T.; Lu, J.; Wang, H., Electrochemical reduction of nitrate to ammonia via direct eight-electron transfer using a copper–molecular solid catalyst. *Nature Energy* **2020,** 5 (8), 605-613.
14. Li, C.; Liu, S.; Xu, Y.; Ren, T.; Guo, Y.; Wang, Z.; Li, X.; Wang, L.; Wang, H., Controllable reconstruction of copper nanowires into nanotubes for efficient electrocatalytic nitrate conversion into ammonia. *Nanoscale* **2022,** 14 (34), 12332-12338.
15. Xu, Y.; Ren, K.; Ren, T.; Wang, M.; Liu, M.; Wang, Z.; Li, X.; Wang, L.; Wang, H., Cooperativity of Cu and Pd active sites in CuPd aerogels enhances nitrate electroreduction to ammonia. *Chemical Communications* **2021,** 57 (61), 7525-7528.
16. Ren, T.; Ren, K.; Wang, M.; Liu, M.; Wang, Z.; Wang, H.; Li, X.; Wang, L.; Xu, Y., Concave-convex surface oxide layers over copper nanowires boost electrochemical nitrate-to-ammonia conversion. *Chemical Engineering Journal* **2021,** 426, 130759.
17. Sun, W. J.; Ji, H. Q.; Li, L. X.; Zhang, H. Y.; Wang, Z. K.; He, J. H.; Lu, J. M., Built-in electric field triggered interfacial accumulation effect for efficient nitrate removal at ultra-low concentration and electroreduction to ammonia. *Angewandte Chemie International Edition* **2021,** 60 (42), 22933-22939.

18. Wei, J.; Ye, G.; Lin, H.; Li, Z.; Zhou, J.; Li, Y.-y., Enhanced electrochemical nitrate reduction on copper nitride with moderate intermediates adsorption. Journal of Colloid and Interface Science **2024,** 670, 798-807.
19. Lu, Y.; Yue, F.; Liu, T.; Huang, Y.-C.; Fu, F.; Jing, Y.; Yang, H.; Yang, C., Size-effect induced controllable Cu0^{-}Cu^{+} sites for ampere-level nitrate electroreduction coupled with biomass upgrading. Nature Communications **2025,** 16 (1), 2392.
